# Determination of absolute intramolecular distances in proteins using anomalous X-ray scattering interferometry†

**DOI:** 10.1039/d4nr03375b

**Published:** 2024-12-09

**Authors:** Samuel Stubhan, Anna V. Baptist, Caroline Körösy, Alessandra Narducci, Gustavo Gabriel Moya Muñoz, Nicolas Wendler, Aidin Lak, Michael Sztucki, Thorben Cordes, Jan Lipfert

**Affiliations:** a Department of Physics and Center for NanoScience, LMU Munich Amalienstr. 54 80799 Munich Germany; b Soft Condensed Matter and Biophysics, Department of Physics and Debye Institute for Nanomaterials Science, Utrecht University Princetonplein 1 3584 CC Utrecht The Netherlands J.Lipfert@uu.nl; c Max Planck Institute of Biochemistry Am Klopferspitz 18 82152 Martinsried Germany; d Physical and Synthetic Biology, Faculty of Biology, LMU Munich Großhadernerstr. 2-4 82152 Planegg-Martinsried Germany cordes@bio.lmu.de; e ESRF 71 Avenue des Martyrs 38043 Grenoble France; f Biophysical Chemistry, Faculty of Chemistry and Chemical Biology, Technische Universität Dortmund Otto-Hahn-Str. 4a 44227 Dortmund Germany; g Institute for Physics, Augsburg University Universitätsstrasse 1 86159 Augsburg Germany

## Abstract

Biomolecular structures are typically determined using frozen or crystalline samples. Measurement of intramolecular distances in solution can provide additional insights into conformational heterogeneity and dynamics of biological macromolecules and their complexes. The established molecular ruler techniques used for this (NMR, FRET, and EPR) are, however, limited in their dynamic range and require model assumptions to determine absolute distance or distance distributions. Here, we introduce anomalous X-ray scattering interferometry (AXSI) for intramolecular distance measurements in proteins, which are labeled at two sites with small gold nanoparticles of 0.7 nm radius. We apply AXSI to two different cysteine-variants of maltose binding protein in the presence and absence of its ligand maltose and find distances in quantitative agreement with single-molecule FRET experiments. Our study shows that AXSI enables determination of intramolecular distance distributions under virtually arbitrary solution conditions and we anticipate its broad use to characterize protein conformational ensembles and dynamics.

## Abstract 2

We establish high-resolution (<1 Å) intramolecular distance measurements in proteins using pairs of small (1.4 nm diameter) gold nanoparticle labels and anomalous small-angle X-ray scattering as a read out.

## Graphical abstract

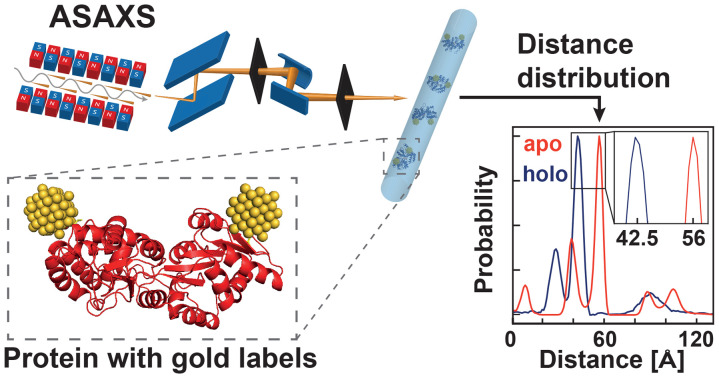

## Introduction

Atomic resolution biomolecular structures are typically determined using frozen or crystalline samples with cryo-EM^1,2^ and X-ray methods,^3–6^ respectively, or in aqueous solution at room temperature with NMR.^7,8^ Measurement of intramolecular distances can provide additional insights into the structure and dynamics of biological macromolecules and their complexes. A sufficient number of intramolecular distances enables the determination of high-resolution structures and can also provide critical information about the conformational ensemble and dynamics of macromolecules based on molecular ruler techniques such as PELDOR/DEER (EPR)^9–11^ or single-molecule Förster resonance energy transfer (smFRET).^12–27^

Also small-angle X-ray scattering can provide information about intramolecular distances in biomolecules. Notably the *P*(*r*) distribution, *i.e.* the Fourier transform of the scattering intensity profile *I*(*q*), is a histogram of pairwise distances and can be readily obtained from scattering data.^28–30^ However, *P*(*r*) does not contain information about which specific pair contributed to a given distance. Labeling macromolecules with electron-rich labels at two positions – *e.g.* heavy atoms,^31–33^ ions, or small gold nanocrystals – combined with SAXS as readout can overcome this limitation. In conventional X-ray scattering interferometry (XSI)^34–40^ with gold labels, the label–label interference term is isolated from other scattering contributions by measuring multiple samples, including the double-labeled, two single-labeled, and unlabeled macromolecule.^34,35^ An alternative approach to separating the gold–gold term, termed anomalous XSI or AXSI, uses anomalous small-angle X-ray scattering (ASAXS) and relies on the energy-dependence of the gold scattering signal.^32,33,41–43^ A regularized Fourier transform of the gold–gold scattering term then directly provides the distribution of distances *P*(*d*) between the gold labels. (A)XSI has several advantages compared to other molecular ruler techniques: (i) it provides distance distributions on an absolute length scale, based on the fact that it is straight-forward to measure the momentum transfer *q* (*q* = 4π sin(*θ*)/*λ*, where 2*θ* is the total scattering angle and *λ* the X-ray wavelength) on an absolute scale; (ii) (A)XSI can provide the full distribution of intramolecular distances (not only mean inter-label distances), without broadening through *e.g.*, photophysics, as seen in FRET or temporal dynamics, due to the short interaction time of X-ray photons with the sample;^34^ (iii) it can readily be applied to distances >10 nm, which remains very challenging for NMR, EPR,^9,10,44^ or FRET;^45^ (iv) finally (A)XSI distance measurements are not sensitive to label orientation or the specific label environment, unlike FRET approaches.^24^ Insensitivity of the distance measurement to the environment is advantageous for measurements to determine conformational changes in response to *e.g.*, denaturant,^46^ salt,^47–49^ or ligand concentration. ASAXS-based AXSI measurements have the advantage that they in principle only require preparation of the double-labeled sample, as opposed to traditional XSI, which requires matching single-labeled constructs as well, which *e.g.* requires expression of additional protein constructs. However, so far AXSI has only been established experimentally for DNA constructs, which can be labeled in a straightforward way.^43^

Here, we demonstrate AXSI intramolecular distance measurements in proteins that undergo conformational changes upon ligand binding. We use MalE, the soluble periplasmic component of the maltose import system of *E. coli*,^50–52^ which has been characterized in detail previously by smFRET experiments and other structural methods.^24,50–57^ MalE undergoes a conformational change from an open/apo to a closed/holo state upon binding maltose with a dissociation constant^24^*K*_d_ of ∼1–2 μM. We analyze AXSI data for two double-cysteine variants of MalE and extract distance distributions of the apo and holo states that exhibit sharp main peaks. The main peak position can be determined with Ångström precision and the measured distances are in good agreement, within experimental error, with quantitative distance determination *via* smFRET.

## Results

### Labeling of MalE mutants *via* thiol–gold chemistry

**Fig. 1:**
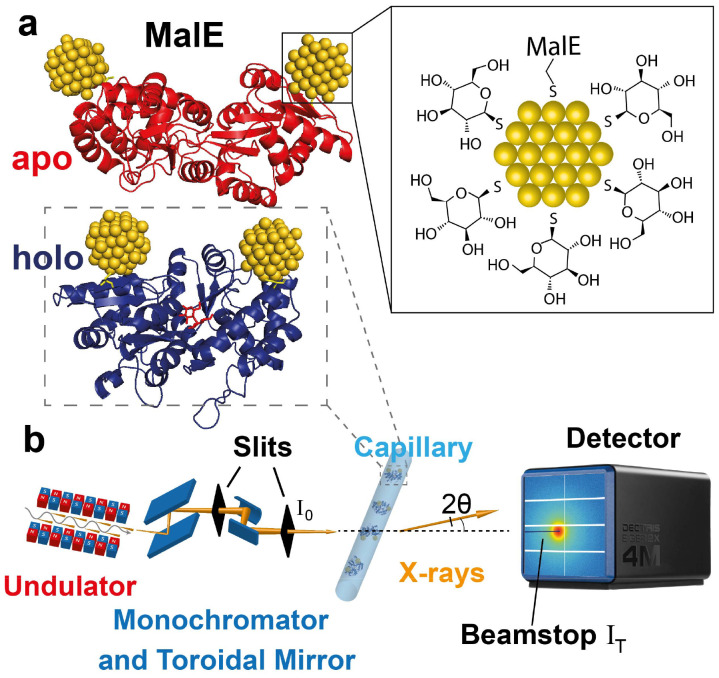
Schematic of anomalous X-ray scattering measurements to determine intramolecular distance distributions. (a) Illustrations of double-labeled MalE in the apo and holo state with gold labels at amino acid positions 36 and 352 (rendered from PDB ID 1OMP (red – apo) and 1ANF (blue – holo), respectively. Gold nanocrystals are positioned using FPS calculations^19^). The zoom depicts the thioglucose shell on the gold NPs as well as the S-Au attachment to the protein. (b) Illustration of the SAXS experiment. The undulator and X-ray optics at the synchrotron beam line provide X-rays with tunable energy. The monochromator is used to select energies and collimated X-rays are scattered by the sample in a quartz capillary. The incident intensity *I*_0_ is measured in front of the capillary and the transmitted intensity *I*_T_ is measured at the beamstop. Scattered photons are collected in an Eiger2X 4M pixel detector.

(A)XSI measurements for computing intramolecular distances necessitate site-specific attachment of gold-labels. For this purpose, we used variants of the 42.4 kDa maltose-binding protein MalE that comprise two cysteines in the two different lobes of the protein (ESI Materials and methods: overexpression, isolation and refolding of MalE proteins†), either at positions 36 and 352 (MalE_31-212_; Fig. 1) or at positions 31 and 212 (MalE_36-352_).^24,52^ The pairs of label positions were chosen to be situated in the different lobes of the protein and use positions that have a high probability of being successfully labeled based on their physico-chemical properties,^58^ but otherwise selected randomly. All four selected positions are at the surface of the protein, but have average B-factors and are in secondary structure elements (residues 31, 212, and 352 are in α-helices and 36 in a β-sheet). MalE lacks native cysteines, thus allowing site-specific attachment of gold labels *via* chemical coupling to thiols^52^ (Fig. 1a). Thioglucose coated gold nanoparticles (NPs) were synthesized following a one-phase Brust–Schiffrin method^59^ (ESI Materials and methods: synthesis of gold nanoparticles†) and exhibit a radius of 0.7 nm with high monodispersity^34,35,40^ (Fig. S1 and S2†). Proteins were labeled with the NP on nickel-sepharose columns^11,52,60^ and subsequently purified by size exclusion chromatography (Fig. S3 and S4†).

### SAXS and ASAXS measurements of MalE constructs

We carried out SAXS measurements at both fixed and variable X-ray energies at beamline ID02 of the European Synchrotron Radiation Facility (ESRF;^61–64^ and ESI Materials and methods: ASAXS measurments†; Fig. 1). Control measurements of the unlabeled protein at fixed energy reveal SAXS profiles indicative of a monodisperse sample and show systematic, but subtle changes upon addition of 10 mM maltose, with radii of gyration in good agreement with predictions from the crystal structures in the open and closed conformations (Fig. S5†).

**Fig. 2:**
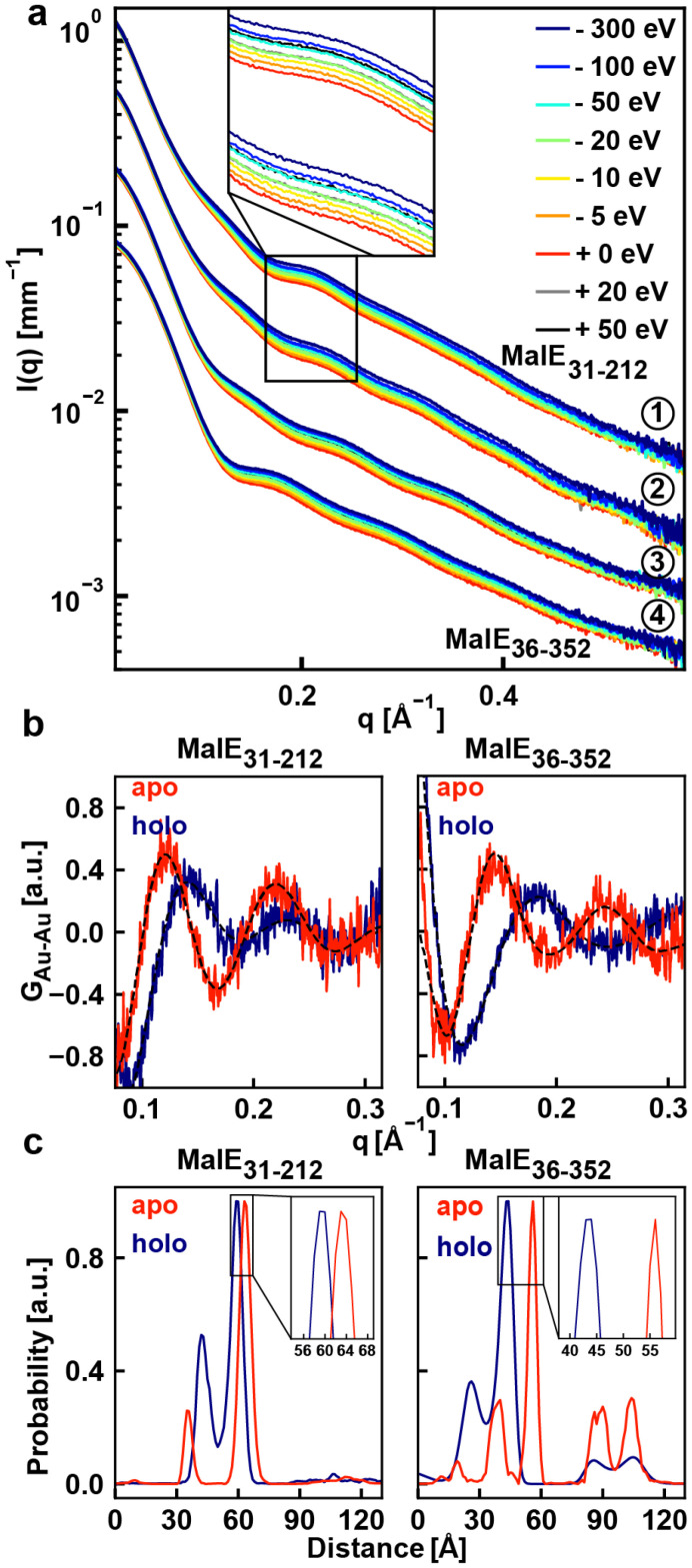
ASAXS data and distance distribution for MalE labeled at position 36 and 352 and MalE labeled at position 31 and 212. (a) ASAXS measurements of both double-labeled MalE mutants with and without maltose measured at 9 energies around the gold L-III absorption edge. ① MalE_31-212_ ② MalE_31-212_ with 10 mM maltose ③ MalE_36-352_ and ④ MalE_36-352_ with 10 mM maltose. Data are vertically offset for clarity (by scaling factors ①:10, ②:6, ③:2 and ④:1). Indicated energies are relative to the Au L-III edge. Alternative representations of the data are shown in Fig. S6.† (b) Gold–gold scattering interference terms for MalE_36-352_ (left) and MalE_31-212_ (right) in the absence and presence of 10 mM maltose after correction for 60% single-labeled and 30% unlabeled contributions. (c) Distance distributions *P*(*d*) obtained by maximum entropy inversion from the interference terms in panel b. The insets show a zoom on the main peaks in the distance distributions.

ASAXS data were recorded for double-labeled, single-labeled, and unlabeled MalE constructs by recording scattering profiles at 9 energies around the gold L-III absorption edge at 11.919 keV (Fig. 2 and Fig. S6†). Ascorbic acid was added to the buffer for all ASAXS measurements to reduce radiation damage for high signal-to-noise measurements (Fig. S7†). The scattering profiles at different energies for the double-labeled MalE_31-212_ and MalE_36-352_ constructs both show oscillations (Fig. 2a), in particular in the range *q* = 0.1–0.2 Å^−1^, which are absent in the unlabeled data (Fig. S5†), indicative of the gold–gold interference contribution.^40^ The oscillations shift upon addition of maltose (Fig. 2a, inset), suggesting a modulation of gold–gold interference term upon addition of maltose. Further, the scattering profiles show systematic changes with X-ray energy: the intensity decreases when approaching the L-III absorption edge.

HPLC purification of the double-labeled sample removes dimers and aggregates from the monomer peak, however, single-labeled and unlabeled species remain in the solution, visible as bands in gel electrophoretic analysis of the sample. From the gel, we estimate ∼60% unlabeled, ∼30%, single-labeled, and ∼10% double-labeled sample, which agrees with a protein : gold NP concentration ratio of 1 : 0.5 (Fig. S3 and S4†). Despite of the relatively low labeling efficiency, we still get a robust signal, since the gold particles scatter strongly with ∼85 atoms and a ∼40 times higher electron density contrast compared to proteins. Additionally, in principle, only the double-labeled sample will contribute a gold–gold term to the scattering pattern. However, the presence of unlabeled and single-labeled species might deteriorate the signal. Since we conducted ASAXS measurements also for single-labeled mutants MalE_31_, MalE_36_, MalE_212_, MalE_352_ and unlabeled samples, we can subtract their scattering contributions from the double-labeled sample (Fig. S8†). We tested and refined the influence of subtracting single- and unlabeled protein contributions in the ASAXS data analysis (see below).

### Determination of the gold label–gold label distance distribution from ASAXS data

We analyzed the ASAXS data and determined the gold–gold scattering contribution from the corrected and energy-dependent scattering data with a matrix inversion approach described previously.^42,43^ The approach takes into account the atomic scattering factor of gold and the form factor of the gold spheres (Fig. S1 and S2†) and exploits the fact that the atomic scattering factors for non-gold atoms show minimal energy dependence within the chosen energy range (Fig. S9†). The matrix inversion yields the gold–gold structure factor *G*_Au–Au_, which was corrected by a constant offset by subtracting the mean^43^ (Fig. 2b) and Fourier transformed with a maximum entropy algorithm^35,40,43,65^ to obtain the real-space distance distributions *P*(*d*) (Fig. 2c).

Using this procedure, we determined the gold label-gold label distance distributions *P*(*d*) for the two MalE variants, MalE_31-212_ and MalE_36-352_, both in absence and presence of a high (10 mM) concentration of maltose (Fig. 2c). Under all conditions, the *P*(*d*) distributions exhibit a major peak and additional, smaller peaks at smaller and larger distances. We find that peaks at smaller and larger distances (>80 Å) are variable from data set to data set and are sensitive to details of the single- and unlabeled subtraction and maximum entropy procedure and are likely due to imperfections of the experimental data^40^ and possibly due to the presence of a small fraction of dimeric samples (Fig. S3†). In contrast, the positions of the main peak in either condition are robust (Tables S1, S2 and Fig. S10†).

### Analysis of uncertainty in AXSI measurements

To determine the uncertainties of the measured distance distributions, we analyzed the variability introduced by several factors. First, we tested the uncertainty introduced by the maximum entropy algorithm used to compute the *P*(*d*) distributions. For each measured gold–gold term *G*_Au–Au_, we carried out 20 repeat runs of the maximum entropy algorithm and fitted the main peak with a Gaussian to determine its position and standard deviation (Fig. S11†). In each run the MemSys5-based^65^ maximum entropy algorithm is randomly selecting and deleting 10% of the scattering data^34^ in contiguous blocks of 2%. We find only small deviations between repeat runs of the maximum entropy algorithm: the main peak positions exhibit standard deviation of 0.2–0.7 Å computed from 20 repeat runs of the maximum entropy algorithm each for different MalE mutants and conditions (Tables S1 and S2†). In addition, we performed control calculation using a different inversion scheme based on Tikhonov regularization (Fig. S12 and S13†).

We again find very similar values (within <1 Å) for the position of the main peak, but a larger width of the central peak and more pronounced secondary peaks at larger and smaller distances.

Next, we test the sensitivity to differences in background subtraction, in particular from subtracting unlabeled and single-labeled contributions to the scattering pattern. We subtracted varying quantities of single-labeled and unlabeled protein contributions from the scattering pattern and computed *P*(d) functions as described above. We find that the secondary peaks in the distance distribution are smallest if 60% unlabeled and 30% single-labeled contributions are subtracted (red curve in Fig. S9† and data shown in Fig. 2) for both mutants MalE_36-352_ and MalE_31-212_, which agrees with the estimated fractions from gel analysis (Fig. S3†). Importantly, adjusting the amount of single and unlabeled contributions subtracted or even using the scattering data without subtraction affects only the level and position of the secondary peaks and changes the main peak position by at most 1 Å (all measured peak positions in Tables S1 and S2†). Thus, our analysis suggests that while the unlabeled and single-labeled contributions add to the level of experimental noise and affect the exact shape of the *P*(*d*) function, the main peak corresponding to the gold label–gold label distance is robust against these perturbations.

Finally, we tested the reproducibility of the AXSI measurement by performing a repeat measurement for each sample. We find that the mean peak position for repeat measurements only varies by at most 0.7 Å (all measured peak positions in Tables S1 and S2†). Taken together, our error analysis suggests that we can determine the center of the main peak in the *P*(*d*) distributions to better than 1 Å (Tables S1 and S2†), in agreement with previous analyses of (A)XSI measurements for nucleic acids.^34,40,43^ As a control, we determined the gold–gold structure factor *G*_Au–Au_ using conventional XSI,^34,35,40^*i.e.* with measurements at only a single X-ray energy of the double-labeled, both single-labeled, and the unlabeled protein samples with SAXS. The control measurement for MalE_36-352_ in the absence of maltose gives a main peak in the distance distributions in excellent agreement with the results of the AXSI analysis (Fig. S14†).

### Determination of MalE intramolecular distances by smFRET

**Fig. 3:**
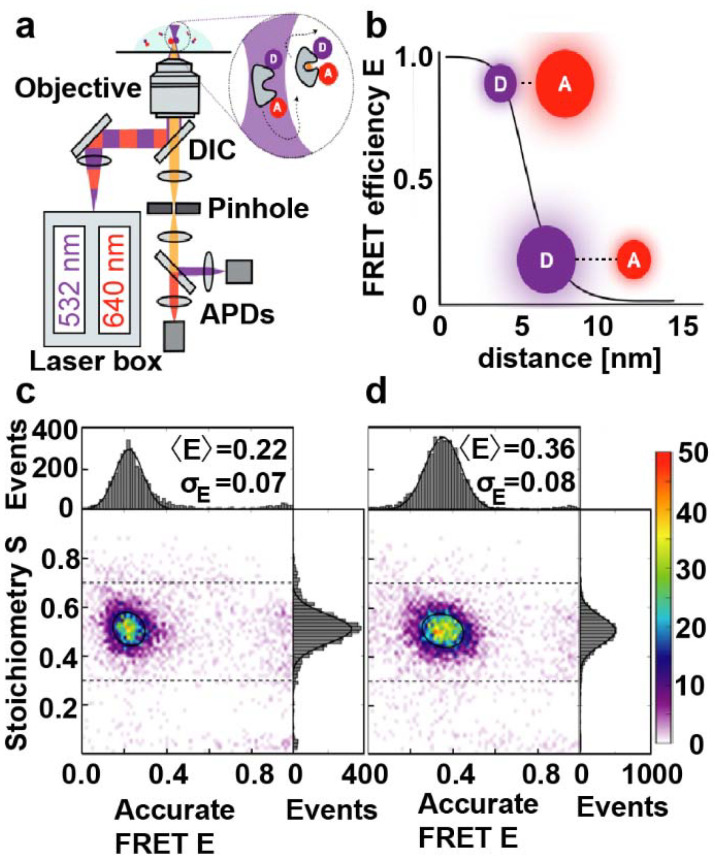
Monitoring conformational changes in MalE by smFRET with ALEX. (a) Schematic overview of an ALEX confocal microscopy setup with two fiber-coupled modulated laser sources for alternating excitation of donor (D) and acceptor (A) dyes. The laser is expanded, collimated, and directed into an objective with a high numerical aperture through a dichroic mirror (DIC). The objective is used for excitation and detection of the fluorescence of individual proteins in a diffraction limited excitation spot. Subsequently, a pinhole spatially filters the fluorescence before it undergoes spectral separation into green and red detection channels. (b) Schematic plot of the FRET efficiency (*E*) as a function of the distance between a donor fluorophore and an acceptor. (c and d) Corrected accurate *ES*-histograms of MalE_31-212_ labeled with Alexa555 and Alexa647 depicting the ligand-free open (c) and the liganded closed (in the presence of 1 mM maltose; (d) conformation.

To provide a reference and enable direct comparison of the results with an established technique, we performed distance measurements on MalE_31-212_ and MalE_36-352_ by single-molecule FRET using alternating laser excitation (ALEX)^66–68^ (Fig. 3a and ESI Methods†). We employed a data analysis approach similar to a recent multi-lab FRET comparison study^24^ to extract mean interprobe distances between donor (Alexa 555) and acceptor fluorophore (Alexa 647) from intensity-based single-molecule measurements^20,22,66–70^ based on the Förster relation (Fig. 3b). Both MalE_31-212_ and MalE_36-352_ are designed to show changes from larger to smaller interprobe distances upon maltose binding. We observe shifts from low to intermediate FRET efficiency values *E* from the apo to holo state, as expected (Fig. 3c, d and Fig. S15†). The mean *E* values change from 0.22 to 0.36 for MalE_31-212_ and from 0.4 to 0.67 for MalE_36-352_, corresponding to a reduction in the calculated interprobe distances (Table 1).

**Table 1:** Intramolecular distances in MalE_31-212_ and MalE_36-352_ in the absence (apo state) and presence of 10 mM maltose (holo state). Values reported for AXSI are the mean ± standard deviation of the peak position averaged over repeat measurements and different levels of background subtractions (all values in ESI Tables S1 and S2†). Values for FRET are the mean ± standard deviation from three technical repeats, using the Förster radius of *R*_0_ = 5.1 nm and quantum yield determined previously for this dye pair.^11^ Errors in the distances from uncertainties in *R*_0_ and quantum yield^11^ are 0.3–0.5 nm and, therefore, smaller than statistical errors from technical repeats. Simulated values are the mean label distances ± the standard deviations of simulated distances. For FRET labels we report the results of the established procedures FPS and FRETraj; the coarse-grained simulations are very similar to the FPS results. For AXSI labels we conversely report values from FPS and the coarse-grained method, since FRETraj gave very similar values to FPS (Fig. 4 and Fig. S16†)

	MalE_31-212_ apo	MalE_31-212_ holo	MalE_36-352_ apo	MalE_36-352_ holo
**Measurements (mean ± error) [Å]**				
AXSI	63.7 ± 0.5	59.4 ± 0.5	56.0 ± 0.3	43.1 ± 0.6
FRET	63.0 ± 2.4	56.3 ± 2.2	54.1 ± 2.1	44.9 ± 1.7
**Simulations (mean ± standard deviation) [Å]**				
FPS AXSI	67.9 ± 0.5	59.7 ± 0.4	60.7 ± 1.2	44.6 ± 0.7
Coarse-grained AXSI	64.4 ± 2.8	56.6 ± 2.0	59.0 ± 4.4	42.7 ± 4.5
FPS FRET	68.6 ± 8.3	56.7 ± 9.5	60.5 ± 11.3	44.3 ± 11.3
FRETraj FRET	63.0 ± 9.6	53.8 ± 10.0	56.8 ± 11.6	42.2 ± 11.8
C_β_ distance	51.5	42.6	50.6	39.8

### Modeling the label geometry for FRET and AXSI measurements

**Fig. 4:**
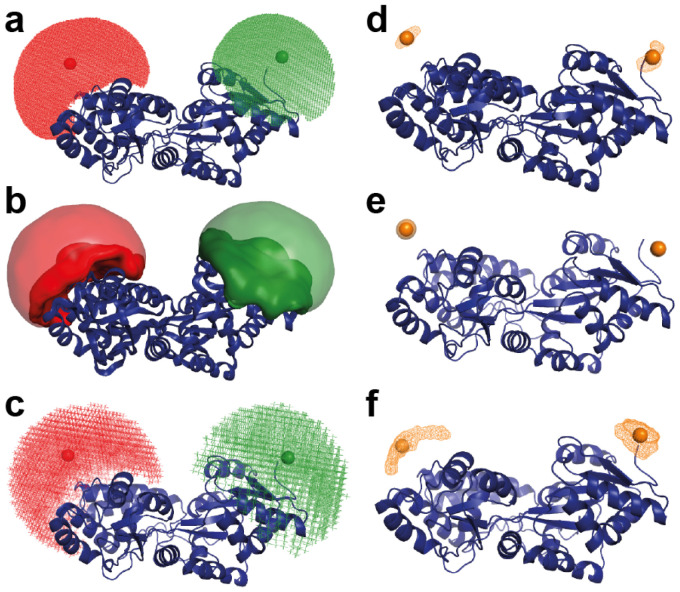
Simulations of label positions for FRET dyes and gold nanoparticles using FPS,^19^ FRETraj,^74^ and a coarse-grained computation of accessible volumes. All panels show MalE_36-352_ in the apo state (PDB ID: 1OMP). (a)–(c) Positions for the FRET dyes Alexa555 and Alexa647 are shown as colored clouds as computed with FPS (a), FRETraj (b), and coarse-grained simulations (c). (d)–(f) Positions of the gold nanoparticles used for AXSI measurements computed with FPS (d), FRETraj (e), and coarse-grained simulations (f). The geometrical parameters used in the calculations are in Table S3.† The resulting mean label–label distances and their standard deviations are in Table 1.

AXSI and FRET give mean distances that are in good agreement (Table 1). However, we note that both AXSI and FRET measure the distances between the respective labels and not directly between the positions of the labeled residues. Therefore, the attachment, size, and flexibility of the organic dyes or gold labels need to be considered when interpreting distance measurements.^20,71^ Taking into account the label geometries is particularly relevant in light of the very high resolution of AXSI measurements, where distances are determined to better than 1 Å (Table 1), which is much smaller than the label and linker sizes and also smaller than the spatial resolution of FRET^24,72^ which is on the order of 2–5 Å. Taking the crystallographic structures of MalE as a starting point, we simulate label positions using label parameters summarized in ESI Table S3† and following several different approaches (Fig. 4). First, we calculated the label distances with FPS (“FRET positioning and screening”),^19,73^ which generates accessible volumes for each dye and computes the distance distribution assuming random sampling of the accessible volume (Table 1, “FPS”). Second, we use FRETraj^74^ to compute distances based on accessible contact volumes (ACVs) (Table 1, “FRETraj”), which have been shown to provide a better estimate of label–label distances than the full accessible volume if the dyes interact with the protein surface.^24^ Finally, we compute accessible volumes using a simple coarse-grained sampling (see ESI Methods†) and calculate the mean and standard deviation of distances in the sampled positions (Table 1, Fig. 4 and Fig. S16†). In all three computational approaches the crystal structure is treated as fixed.

To model the label positions for AXSI measurements, we adopted the same procedures considering the size and attachment of the gold nanoparticles. The gold nanoparticles, in contrast to fluorescent dyes, are directly attached to the sulfur atom of the cysteine residues. Therefore, the C_β_–Au distance is only ∼3 Å. The attached gold NP have a radius of 7 Å, resulting in a distance from attachment atom C_β_ to the center of the label of ∼10 Å. The much more confined attachment of the gold nanoparticles compared to the fluorescent dyes used for FRET results in much more narrow predicted distance distributions from the modeling of the label and its linker: the predicted distributions of label positions for FRET labels have standard deviations of ∼10 Å in contrast to only ∼0.5–4 Å for our AXSI labels (Table 1).

### Comparison of distances from AXSI and FRET and modeling

**Fig. 5:**
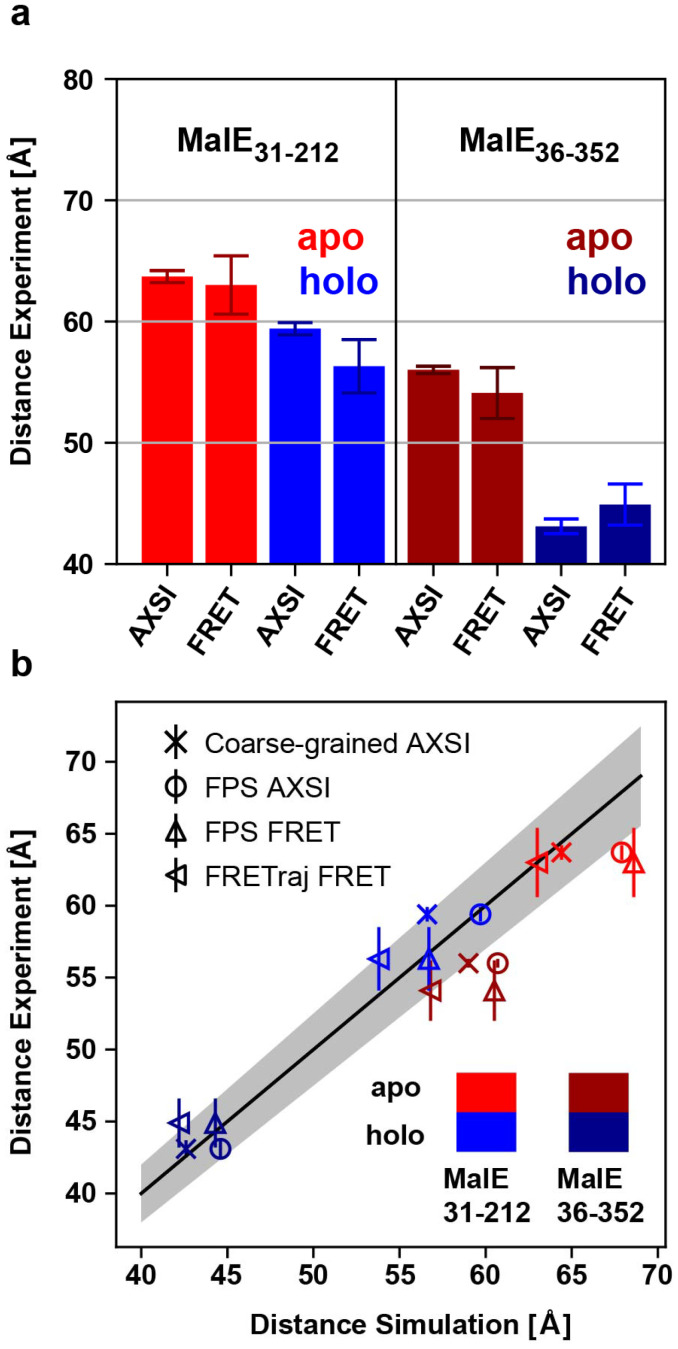
Comparison of AXSI and FRET measurements and structural modeling. (a) Experimentally determined distances from AXSI and FRET for both MalE variants. Errorbars depict experimental errors (see main text). (b) Comparison of experimentally determined distances and the structural models: coarse-grained for AXSI labels (crosses), FPS for AXSI labels (circles), FPS for FRET labels (upward triangles) and FRETraj for FRET labels (left triangles). Experimental uncertainties of the respective techniques are shown. The solid line marks a 1 : 1 relation and the grey area indicates 5% deviation.

The intramolecular distances determined experimentally by AXSI and FRET agree for MalE_31-212_ and MalE_36-352_ in both the apo and holo state within error (Table 1 and Fig. 5). This close agreement, despite the different labels used, suggests that the different physico-chemical properties and their differences in geometry do not significantly affect the mean positions of the labels and do not bias or perturb the conformations of the protein. Comparing the experimentally determined distances to the modeled distance distributions, we find good agreement of the mean distances in the closed or holo state for both MalE_31-212_ and MalE_36-352_. In the closed state, all of the approaches to model the mean distances give fairly similar results, in particular for MalE_36-352_, due to the direction of the label attachment being approximately perpendicular to the vector connecting the label centers (Fig. 1a). In contrast, in the open or apo state the predicted distances tend to be larger than the experimentally determined values, in particular for MalE_36-352_ (Fig. 5). We note that for the open conformation the details of the modeling play a larger role, compared to the closed conformation. In particular, the predictions based on the accessible surface volume using FRETraj fit the experimental data for FRET better than the accessible volume-based predictions, suggesting that the fluorescent labels might have some tendency to stick to the protein's surface.^24,73^ The differences in the apo state might also indicate that the protein conformations in solution could deviate from the crystal structure. As a plausibility test, we used a simple elastic network model approach^75^ to deform the structure of the open conformation of MalE (Fig. S17†) and find that a simple deformation of the protein along the first normal mode could explain the observed difference between measured and predicted distances.

Unlike in FRET measurements, AXSI provides, at least in principle, the full distance distribution.^34,43^ The mean peaks of the *P*(*d*) distributions are fairly narrow, with a width of typically ∼2–5 Å (“peak width”-column in Tables S1 and S2†), which is similar to the distributions obtained by modeling the relatively inflexibly attached gold nanoparticles, assuming otherwise static protein structures, suggesting that the labeled residues of MalE and adopt a fairly static conformation in solution, as might be expected for a well-folded, globular protein. However, we note that the width of the *P*(*d*) is less well defined and more difficult to determine from the experimental data than the main peak position, suggesting that further improvements of the method are necessary to accurately determine and interpret the full molecular ensemble. In particular, a higher labeling efficiency would be desirable, in particular since previous (A)XSI measurements on nucleic acids with a high labeling efficiency have successfully provided full distance distribution information.^43^

## Discussion

In summary, we demonstrate accurate intramolecular distance measurements using AXSI for a protein that undergoes ligand-induced conformational motion. Mean distances can be determined very precisely (within <1 Å) and we find excellent agreement with distances measured experimentally by quantitative FRET. The good agreement between FRET, AXSI, and modeled structures strongly suggest that the MalE constructs used in this work are not significantly perturbed or denatured by our labeling approach, consistent with the observation that the binding affinity of labeled MalE agrees closely, within experimental error, with measurements on wildtype MalE^24,50,52,53,76^ (Fig. S18†). In the future, improved labeling and purification procedures and more sophisticated modeling approaches, *e.g.* based on molecular dynamics simulations, should enable improved estimates of the full distance distributions and allow for full quantitative comparisons to molecular models. A range of different labeling and functionalization approaches is also desirable to select functionalization approaches that minimally perturb the protein of interest.^77–80^ Simulations for distributions with the same mean but different widths suggest that even considerably broader ensembles should be resolvable using AXSI (Fig. S19†). In addition, (A)XSI has the potential to discern different sub-populations in the *P*(*d*) distribution, as has been demonstrated for a Holliday junction using XSI.^81^ Simulation with different size particles suggest that in principle smaller gold nanoparticles could be used, however the signal-to-noise deteriorates with decreasing gold nanoparticles (Fig. S20†).

## Conclusions

The introduction of AXSI for proteins opens up exciting possibilities in structural biology and beyond. The ability to determine intramolecular distance distributions for proteins in free solution and under virtually arbitrary solution conditions has the potential to address questions regarding partially folded conformations and natively disordered proteins.^46,82–84^ In principle, our method could be used on dehydrated or frozen samples^85^ and our labeling approach might be adapted for marking structures in cryo-EM measurements.^86,87^ Beyond intramolecular distance measurements, AXSI might be extended to studying protein–protein interactions, *e.g.* by monitoring inter-molecular label–label distances in complexes or even assemblies of single-labeled proteins. In addition, our approach might be used to probe ligand binding kinetics and signaling pathways. Finally, the integration of computational modeling could establish a platform for refining structural predictions.

## Author contributions

T.C. and J.L. designed the study; A.N., G.G.M.M., and N.W. prepared protein samples; S.S., A.V.B., C.K., and A.L. prepared gold-labeled samples; S.S., A.V.B., C.K., and M.S. performed ASAXS measurements; A.N., G.G.M.M., and N.W. performed FRET measurements; S.S., A.V.B., and G.G.M.M. analyzed the data; T.C. and J.L. supervised research; S.S., T.C., and J.L. wrote the manuscript with input from all authors.

## Data availability

Data for this article, including ASAXS scattering profiles, gold label–gold label interference terms and distance distributions, are available in the public repository YODA at https://doi.org/10.24416/UU01-CKHVKZ.

## Conflicts of interest

The authors declare no competing financial interest.

## Supplementary Material

NR-017-D4NR03375B-s001
