# Supplementary material for: Determination of absolute intramolecular distances in proteins using anomalous X-ray scattering interferometry
Source: Nanoscale. 2024 Dec 9;17(6):3322–30. doi: 10.1039/d4nr03375b (PMC11653172; doi:10.1039/d4nr03375b)
Supplement: NR-017-D4NR03375B-s001 [file NR-017-D4NR03375B-s001.pdf]

Supplementary Information (SI) for Nanoscale.
This journal is © The Royal Society of Chemistry 2024

SUPPLEMENTARY INFORMATION FOR

Determination of Absolute Intramolecular Distances in Proteins by Anomalous X-ray Scattering Interferometry

Samuel Stubhan[1,2], Anna V. Baptist[1,3], Caroline Körösy[2], Alessandra Narducci[4], Gustavo Gabriel Moya Muñoz[4,6], Nicolas Wendler[4,6], Aidin Lak[1], Michael Sztucki[5], Thorben Cordes[4.6,*], Jan Lipfert[1,2.*]

[1]*Department of Physics and Center for NanoScience, LMU Munich, Amalienstr. 54, 80799 Munich, Germany*

[2]*Soft Condensed Matter and Biophysics, Department of Physics and Debye Institute for Nanomaterials Science, Utrecht University, Princetonplein 1, 3584 CC Utrecht, The Netherlands*

[3]*Max Planck Institute of Biochemistry, Am Klopferspitz 18, 82152 Martinsried, Germany*

[4]*Physical and Synthetic Biology, Faculty of Biology, LMU Munich, Großhadernerstr. 2-4, 82152 Planegg-Martinsried, Germany*

[5]*ESRF, 71 Avenue des Martyrs, 38043 Grenoble, France*

[6]*Biophysical Chemistry, Faculty of Chemistry and Chemical Biology, Technische Universität Dortmund, Otto-Hahn-Str. 4a, 44227 Dortmund, Germany*

[7]*Institute for Physics, Augsburg University, Universitätsstrasse 1, 86159 Augsburg, Germany*

[*]Correspondence: cordes@bio.lmu.de; J.Lipfert@uu.nl

CONTENT

Supplementary Materials and Methods
Tables S1-S3
Figures S1-S20

Supplementary Materials and Methods

Overexpression, isolation and refolding of MalE proteins.

Each single cysteine variant (MalE T31C, I212C, T36C, and S352C) and double variant (MalE T31C/I212C and MalE T36C/S352C) of MalE protein was expressed and purified according to published procedures[1]. The T7/lac bacterial expression vector pET-23b(+) carrying a C-terminus 6x-His Tag sequence and containing the DNA coding sequence of MalE wild-type protein[2] was used as the template to generate the cysteine variants via PCR site-directed mutagenesis. *E. coli* BL21(DE3)pLysS transformant cells were grown at 37°C in LB medium containing ampicillin (0.1 mg/ml) and chloramphenicol (0.05 mg/ml). When an optical density (OD600) of 0.6-0.8 was reached, protein overexpression was induced by addition of 0.25 mM isopropyl β-D-1-thiogalactopyranoside (IPTG). The cells were harvested after 1.5 h and resuspended in 50 mL lysis buffer (50 mM Tris-HCl, pH 8.0, 1 M KCl, 10% glycerol, 10 mM imidazole and 1 mM dithiothreitol) for 2 L harvesting culture. Prior to cell disruption, cells were incubated for 30 minutes at 4°C in the presence of DNase I 500 ug/ml, cOmplete™, EDTA-free Protease Inhibitor Cocktail (1 tablet for each 50 mL bacterial culture), 0.2 mM phenylmethylsulfonyl fluoride (PMSF), and 2 mM dithiothreitol (DTT). Cells were lysed using an ultrasonic homogenizer (Branson Digital Sonifier; 25% amplitude, total exposure time to ultrasound of 10 minutes, with time lapses of 0.5 s ON/OFF pulse switches). Cell debris were discarded by a first step centrifugation at 5,000 × *g* for 30 min at 4°C. Next, the cell lysate supernatant was collected by a further ultracentrifugation step at 208,400 x *g* for 1 hour at 4°C. Each MalE protein was purified by Immobilized Metal Affinity Chromatography (IMAC) using the Ni Sepharose® 6 Fast Flow resin pre-washed with 10 column volumes lysis buffer. The cell lysate supernatant was loaded onto Ni^{2+}-sepharose resin (50 mL supernatant for 4 mL resin wet volume) and incubated overnight at 4°C. After two subsequent washing steps with 10 CV lysis buffer supplemented with 5 mM β-mercaptoethanol (BME) and 0.2 mM PMSF, the 6x-His Tagged MalE protein was eluted with 5 CV elution buffer (50 mM Tris-HCl, pH 8.0, 50 mM KCl, 10% glycerol, 250 mM imidazole, 0.2 mM PMSF and 5 mM BME). The eluted protein was dialyzed overnight at 4°C against 250 volumes of dialysis buffer (50 mM Tris-HCl, pH 8.0, 50 mM KCl and 5 mM DTT) to eliminate the excess of imidazole and using the SnakeSkin 10 kDa MWCO dialysis membrane tubing.

After the overnight dialysis at 4°C, the freshly purified MalE proteins were directly subjected to unfolding and refolding processes prior to storage, using adapted procedures based on Ganesh *et al.*[3]. The isolated MalE variants were diluted down to 10-20 µM final concentration in unfolding buffer (6 M final concentration guanidine hydrochloride in 10 mM HEPES, pH 7.3). Each unfolding mixture was incubated for 3 hours under gentle rocking at 30°C. The unfolded protein solutions were afterwards centrifuged at 3046 *g* for 45 minutes at 4°C to remove irreversible aggregates that could hinder correct protein refolding. In parallel, a SnakeSkin 10 kDa MWCO dialysis membrane tubing was pre-soaked in water at 4°C for 1-2 min. The protein refolding was conducted at 4°C by a two-step dialysis process and over a total incubation period of 2 days. First, the proteins were each dialyzed against 100 volumes of refolding buffer (10 mM HEPES, 150 mM NaCl, pH 7.3) supplemented with 200 mM L-arginine. After 18-20 hours, a second dialysis step was carried out by exchanging the dialysis

buffer solution with further 100 volume excess of refolding buffer. Both dialysis steps were conducted in the presence of 5 mM DTT. The refolded MalE proteins were collected after 42 hours and each concentrated to 500 µL final volume (starting from 2 L bacterial culture) using the Vivaspin centrifugal concentrator 10 kDa MWCO. The protein concentration was conducted at 3000 *g* at 4°C. The concentrated proteins were further loaded onto the Superdex™ 75 Increase 10/300 GL column using the automatized ÄKTA pure™ chromatography system (formerly GE Healthcare). For each protein, the column was pre-equilibrated with 2 CV storage buffer (labeling buffer I: 50 mM Tris-HCl, pH 7.4, 50 mM KCl supplemented with 2 mM DTT). Lastly, the MalE variants were concentrated to final concentration between 300 µM and 500 µM and finally stored at -80°C until further use. The final protein concentrations were determined with a NanoPhotometer® N60/N50 (IMPLEN), using the molar extinction coefficient of MalE (66350 $M^{-1} \cdot cm^{-1}$).

FRET labelling and purification of MalE variants $MalE_{31\text{-}212}$ and $MalE_{36\text{-}352}$.
Generally, we followed an already established protocol for stochastic maleimide labelling and purification of MalE proteins[1, 2, 4]. His_6-tagged MalE double-cysteine variants were incubated in labelling buffer (50 mM Tris-HCl pH 7.4, 50 mM KCl) supplemented with 1 mM dithiothreitol (DTT) to retain the reduced state of both cysteine residues. Subsequently, the MalE variants were immobilized by nickel affinity on a nickel functionalized agarose medium (Ni^{2+}-Sepharose™ 6 Fast Flow, Cytiva). After that, the maleimide reaction with 50 nmol of each Alexa Fluor 555 (ThermoFisher) and Alexa Fluor 647 (ThermoFisher) was carried out in labelling buffer overnight at 4°C. The labeled, resin-bound proteins were washed with one column volume (void volume) labelling buffer and eluted with 500 µl labelling elution buffer (50 mM Tris-HCl pH 8.0, 50 mM KCl, 500 mM imidazole). Following the maleimide labelling with Alexa Fluor 555 and Alexa Fluor 647, the raw, fluorophore-labeled proteins were purified by size-exclusion chromatography (ÄKTA pure™ chromatography system, Cytiva; Superdex™ 75 Increase 10/300 GL, Cytiva) or anion exchange chromatography (ÄKTA pure™ chromatography system, Cytiva; MonoQ™ 5/50 GL column, Cytiva), respectively.
The eluate from the maleimide labelling protocol was prepared for further purification by removal of the remaining KCl and imidazole from the labelling elution buffer that could otherwise interfere with the anion exchange process. This step was done using a Sephadex G-25 medium (PD MiniTrap™ G-25, Cytiva) column. The labeled protein was then eluted in 1 ml of anion exchange sample buffer (10 mM Tris-HCl pH 7.5). The anion exchange column was set up with a 5 CV (resin volume) ddH_2O wash, a 10 CV (resin volume) equilibration with anion exchange sample buffer, 10 CV (resin volume) equilibration with anion exchange elution buffer (10 mM Tris-HCl pH 7.5, 1 M NaCl) and a final 20 CV (resin volume) equilibration with anion exchange sample buffer. The washing step and all equilibrations were performed at 1 ml/min flow rate. Labeled protein was loaded onto the column with 0.5 ml/ min and the resin-bound protein was subsequently washed with 10 CV anion exchange sample buffer at 1 ml/min flow rate. For the consecutive elution a linear increase in anion exchange elution buffer ratio with a slope corresponding to 7.5 mM NaCl per column volume was chosen. The flow rate was adjusted to 0.5 ml/ min. Fractions containing the Alexa Fluor 555 and Alexa Fluor 647 labeled protein were selected and used for further analysis.

smFRET measurements and data analysis

Solution-based single-molecule FRET experiments were conducted on a homebuilt ALEX confocal microscope, as described in Kapanidis et al.[5]. All samples were measured in a 100 µL PBS droplet with a protein concentration ranging from 50 to 100 pM on a coverslip passivated with BSA (1 mg/ml in PBS). The experimental setup utilizes alternating laser excitation (ALEX) of two diode lasers: OBIS 532-100-LS (Coherent, USA), operated at 60 µW for donor molecules at 532 nm, and OBIS 640-100-LX (Coherent, USA), operated at 25 µW for acceptor molecules at 640 nm, both in alternation mode with a 100 µs total alternation period (20 kHz frequency). The lasers were combined by an aspheric fiber port (PAF2S-11A, Thorlabs, USA), coupled into a polarization-maintaining single-mode fiber P3-57 488PM-FC-2 (Thorlabs, USA), collimated (RC12APC-P01, Thorlabs, USA), before entering an epi-fluorescence confocal microscope (Olympus IX71, Hamburg, Germany), and guided into a water immersion objective (60X, NA 1.2, UPlanSAPO 60XO, Olympus, Japan) by a dual-edge beamsplitter ZT532/640rpc (Chroma/AHF, Germany). Fluorescence emitted from the sample was collected by the same objective and spatially filtered using a pinhole with a 50 µm diameter and spectrally split into the donor and acceptor channels by a single-edge dichroic mirror H643 LPXR (AHF, Germany). Fluorescence emission was filtered (donor: BrightLine HC 582/75 (Semrock/AHF, Germany); acceptor: Longpass 647 LP Edge Basic (Semrock/AHF, Germany) and focused onto avalanche photodiodes (SPCMAQRH-64, Excelitas, Canada). The detector outputs were recorded by an NI-Card PCI-6602 (National Instruments, USA) using LabView data acquisition software of the Weiss laboratory as outlined previously[6].

Data analysis was performed using a home-written software package, as described in [4]. Bursts corresponding to single-molecule transits through the confocal volume were identified first using an all-photon burst-search (APBS) with a threshold of 15, a time window of 500 µs, and a minimum total photon number of 150 to determine donor leakage crosstalk (α) and direct acceptor excitation (δ). Then, a Dual-Channel-Burst-Search (DCBS) was performed with similar parameters to determine excitation flux (β) and detection efficiency and quantum yields differences (γ) to obtain accurate FRET values E as described[7] .

E histograms of double-labeled FRET species with Alexa Fluor 555 and Alexa Fluor 647 were extracted by selecting $0.3 < S < 0.7$ for 1D-E projections. *E* histograms of the open state without ligand (apo) and the closed state with 1 mM maltose (holo) were fitted with a Gaussian distribution $A \cdot e^{-\frac{(E-\mu)^2}{2\sigma^2}}$. Distance conversion was done according to $R = R_0 \sqrt[6]{(1-E)/E}$, where R0 is the Förster radius[8] given by:

$$R_0{}^6 = \frac{9\,ln(10)}{128\pi^5\,N_A}\frac{\kappa^2}{n^4}Q_D\frac{\int_0^\infty F_D(\lambda)\varepsilon_A(\lambda)\lambda^4 \mathrm{d}\lambda}{\int_0^\infty F_D(\lambda)\mathrm{d}\lambda}$$

Here, NA is the Avogadro constant, κ2 the dipole orientation factor, n the averaged refractive index of the medium, QD the donor quantum yield, FD the donor emission spectrum, and the acceptor absorbance spectrum ε_A. The Förster radius for Alexa Fluor 555 – Alexa Fluor 647 was R0 = 51Å according to [1]. The uncertainties in accurate FRET efficiencies were estimated

through the error propagation of the γ factor, with a relative uncertainty of $\Delta\gamma/\gamma$ = 23% as described in[7].

Synthesis of gold nanoparticles

Gold nanoparticles were synthesized as previously described[9]. Briefly, 0.54 g of gold(III) chloride hydrate and 1 g of β-D-thioglucose were dissolved in 72 ml of a 5:1 methanol:acetic acid solution (concentrations: 22 mM Gold(III) chloride and 63.5 mM β-D-thioglucose). The solution was stirred for 20 min at room temperature. Then, 0.9 g of sodium borohydride were dissolved in 20 ml distilled water (1.19 M sodium borohydride solution). The solution was added dropwise using a dropping funnel to the thioglucose-gold solution over 15 min with constant flow rate while stirring. The solution was stirred for another 30 minutes. Afterwards the solution (~90 ml) is concentrated to 10-15 ml using a rotary evaporator with 10 mbar at room temperature (~30-45 min). The rotation speed was set to 100 rotations per minute. Aggregates were filtered out using 0.45 µm CME filter units and the solution was desalted using an HPLC system with a Sephadex™ G25 column. The final glucose-coated gold nanoparticles are dissolved in ddH_2O and are stable for weeks at 4 °C or for months at −20°C. The protocol yields approximately 2 ml of gold nanoparticle at ≈2 mM concentration, which is sufficient for 3-5 protein labeling runs.

Protein–gold nanoparticle conjugation and purification

Maltose binding proteins (MBPs) were conjugated to the gold nanoparticles synthesized as described in the previous section. The His-tags of the proteins were immobilized on a fast flow nickel sepharose column. Binding the proteins on nickel sepharose resin during labeling, reduces aggregation and improves the labeling efficiency. The column, containing the protein (protein solution: 500 µl with 100 µM), was flushed with 50 mM Tris-KCl buffer, pH 8. The gold nanoparticles were buffer exchanged to the same buffer with Amicon® centrifugal filters (3kDa MWCO) and introduced to the column (500 µl with 2000 µM). Importantly, the molar concentration of gold was at least 20-fold higher than the protein concentration to improve the labeling efficiency and reducing aggregation. The column was sealed and left undisturbed at 4 °C over night. The excess gold nanoparticles were flushed out with 50 mM Tris-KCl buffer and reused. The labeled proteins were eluted from the column and collected with 50 mM Tris-KCl buffer supplemented with 250 mM imidazole. The collected proteins were subsequently purified using an HPLC system with a Superdex™ 75 column and we selected the fraction with the highest labeling efficiency by the A260/A280 ratio and absorption at 360 nm (procedure and chosen fractions are shown in Supplementary Figure S2 and S3). To prepare sufficient quantities for (A)SAXS measurements, multiple column runs were performed. After purification, the selected fractions were pooled and concentrated with Amicon® centrifugal filters with a 3 kDa MWCO.

ASAXS measurements

ASAXS measurements were performed using a flow-through capillary cell with a diameter of 2 mm at the beamline ID02 of the European Synchrotron Radiation Facility, Grenoble (France) using a sample to detector distance of 1.2 m[10]. 2D scattering images were recorded using an Eiger2X 4M detector at 9 energies (11619, 11819, 11869, 11899, 11909, 11914, 11919, 11939, 11969 eV) around the L-III absorption edge of gold to over-determine the matrix equation (see below). 2D data were reduced to azimuthally averaged 1D scattering

profiles using standard techniques. To achieve highest accuracy, the normalization to absolute units in [mm^{-1}] was repeated at each energy and for each sample using a second water-filled flow-through capillary[11, 12]. For each measurement, ≈30 µl of the gold labeled protein samples with a concentration of ≈100 µM (≈ 4 mg/ml) were loaded in to the capillary using a syringe pump and moved during the experiment to avoid radiation damage. After ramping the X-ray energy over the absorption edge, the measurement at 11619 eV was repeated and compared with the first recording to exclude a deterioration of the sample during the ASAXS measurements.
Matching buffer solutions for background correction were measured using the same flow-through cell after concentration with amicon filters at the same X-ray energies. In this way, the buffer scattering could be subtracted from the sample scattering with highest accuracy before further analysis. A single AXSI measurement with 9 different energies takes ≈10-20 minutes depending on the exposure time settings, since for every measurement a water filled capillary and an empty capillary is measured for reference.

ASAXS data analysis

The atomic scattering factor close to an atomic absorption edge is well described by:

$$f(E) = f_0 + f'(E - E_0) + if''(E - E_0)$$

Away from an absorption edge the f' and f'' terms are negligible and the atomic scattering factor is given by f_0. At an absorption edge the energy of the incident photon is equal to an electronic transition. For gold at 11.919 keV one electron from the L-III orbit ($2p_{3/2}$ -electron) is ejected from the atom and the photon is absorbed. Therefore, close to the absorption edge, the *f'* and *f''* terms increase and the scattering factor becomes anomalous.
To perform ASAXS with gold labels we measure at 9 different energies around the L-III absorption edge. At the edge the fraction of absorbed photons by gold is larger and less photons are scattered than off-edge. The scattering from other atoms remains approximately unchanged. Here we use a matrix inversion approach[13, 14] to isolate the partial structure factor of the gold-gold interference from other scattering contributions..
The scattering profiles of the 9 selected energies are normalized as described in the supplementary information of[13] using the theoretical intensity scaling factors for gold given by:

$$int(E) = |f_{Au}(E)|^2 + 2Re(f_{Au}(E))$$

ASAXS data reduction was carried out similar to Pinfield and Scott[14] and Zettl *et al.*[13]. Briefly, we determined the gold-gold scattering contribution from the corrected and energy-dependent scattering data with a matrix inversion approach based on the following matrix relation:

$$\begin{pmatrix} I_{E_1}(q) \\ I_{E_2}(q) \\ \vdots \\ I_{E_{N_E}}(q) \end{pmatrix} = \begin{pmatrix} a_{E_1} & b_{E_1} & c_{E_1} \\ a_{E_2} & b_{E_2} & c_{E_2} \\ \vdots & \vdots & \vdots \\ a_{E_{N_E}} & b_{E_{N_E}} & c_{E_{N_E}} \end{pmatrix} \times \begin{pmatrix} G_{Au-Au}(q) \\ G_{Au-prot}(q) \\ G_{prot-prot}(q) \end{pmatrix}$$

$$I = T \times G$$

Here the scattering intensity vector I has the scattering profiles for all energies concatenated with dimensions $(N_E \times N_q, 1)$, where N_E is the number of energies and N_q is the number of measured q channels. The transformation matrix T consists of the diagonal submatrices a, b and c with dimensions (N_q, N_q), where

$$a_{E_i} = |f_{Au}(E_i)|^2 \cdot F_a(q)$$

with f_{Au} being the atomic scattering factor of gold and F_a is the squared form factor of the gold spheres calculated with the measured radius distribution of the gold labels. Similarly, we have:

$$b_{E_i} = f_{Au}(E_i) \cdot f_{prot} \cdot F_b(q)$$

and

$$c_{E_i} = |f_{prot}|^2 \cdot F_c$$

With the atomic scattering factor of the protein:

$$f_{prot} = \frac{1}{N_{atoms}} \cdot (N_H \cdot f_H + N_C \cdot f_C + N_N \cdot f_N + N_O \cdot f_O + N_S \cdot f_S)$$
$$= \frac{1}{5742}(1852 \cdot f_H + 2865 \cdot f_C + 469 \cdot f_N + 548 \cdot f_O + 8 \cdot f_S)$$

where the atomic composition of the MBP mutant $MalE_{36\text{-}352}$ was used. F_b is the form factor of the gold spheres and F_c is a unit matrix since the protein form factor is neglected. Scattering factor data is taken from:
http://skuld.bmsc.washington.edu/scatter/AS_periodic.html

We can disregard the energy dependence of the protein scattering factor in our case, as atomic scattering factors for non-gold atoms in the sample show minimal energy dependence within the chosen energy range (Figure S7). Lastly, the G vector $(3 \times N_q, 1)$ contains partial structure factors of gold-gold (G_{Au-Au}), gold-protein ($G_{Au-prot}$) and protein-protein ($G_{prot-prot}$). The matrix I is experimentally determined from ASAXS measurements of double-labeled samples, while the transfer matrix T is computed using tabulated atomic scattering factors and treating the gold labels as spheres with a radius distribution around 7 Å (Supplementary Figure S1). Matrix inversion then yields the matrix G, which contains the gold-gold structure factor. The q-range was cut to 0.077 $Å^{-1}$ to 0.315 $Å^{-1}$ to focus on the oscillation. The gold-gold structure factor G_{Au-Au} was corrected by a constant offset by subtracting the mean and transformed with a maximum entropy algorithm to obtain the pair distance distributions P(d). The MemSys5 based[15] maximum entropy algorithm is using a bootstrapping approach, where 10 % of the scattering data are randomly selected and removed in 5 contiguous blocks of 2 %. The remaining 90 % are employed to compute the distance distribution[9].

Subtraction of unlabeled and single-labeled proteins

Achieving high sample purity proves challenging for our gold-labeled proteins. The double-labeled sample also contains single-labeled and unlabeled proteins even after purification. To account for the presence of single-labeled and unlabeled proteins, we subtracted different amounts of single-labeled and unlabeled profiles from the scattering curves of the double-labeled sample. Protein gels give a first estimate for the ratio of double-labeled to single-labeled to unlabeled proteins as 10% to 30% to 60% (Figure S2). We chose single-labeled mutants MalE352 and MalE31 for the subtraction. Importantly, we note that subtracting different amounts of unlabeled or single-labeled protein is not shifting the main peak position for more than 1 Å. (Figure S10, S11 and Table S1, S2). We find that subtracting 60% of unlabeled and 30% of single-labeled sample minimizes secondary peaks. We used Guinier analysis[16] to scale AXSI measurements of single-labeled sample and unlabeled sample to match the concentration of the double-labeled sample, where we used the following values:

$$\rho_{solvent} = 0.334 \frac{e}{\text{Å}^3}$$
$$\rho_{gold} = 4.128 \frac{e}{\text{Å}^3}$$
$$\rho_{prot} = 0.422 \frac{e}{\text{Å}^3}$$

where ρ is the electron density of solvent, gold and the protein. ρ_{gold} was calculated from the scattering length density at 11619 eV using an online calculator http://www.refcalc.appspot.com/sld. The electron densities for protein and solvent were taken from CRYSOL[17]. Furthermore, we also needed the volumes of the protein and the gold-spheres:

$$V_{gold} = 1437\ \text{Å}^3$$
$$V_{prot} = 59.230\ \text{Å}^3$$

For the double-labeled sample, the forward scattering is proportional to:

$$I(0) \sim (\Delta\rho_{prot} \cdot V_{prot} + 2 \cdot \Delta\rho_{gold} \cdot V_{gold})^2.$$

For single-labeled proteins:

$$I(0) \sim (\Delta\rho_{prot} \cdot V_{prot} + \Delta\rho_{gold} \cdot V_{gold})^2.$$

And for unlabeled proteins:

$$I(0) \sim (\Delta\rho_{prot} \cdot V_{prot})^2.$$

Combined, we obtain the following $I(0)$ ratios for monodisperse samples with identical concentration:

$$I(0)_{unlabeled} = 0.105\, I(0)_{double}$$

$$I(0)_{single} = 0.44\, I(0)_{double}$$

Additionally, we computed the forwardscattering from pdb files that contained MalE and modeled gold nanocrystals with CRYSOL[17], which improves the calculation by taking the solvation shell into account.

$$I(0)_{unlabeled} = 0.12\, I(0)_{double}$$

$$I(0)_{single} = 0.45\, I(0)_{double}$$

The scaling factors above are then applied to our assumed labeling efficiencies (from gel electrophoresis and Äkta runs): 60% unlabeled and 30% single labeled in the double-labeled sample and 60 % unlabeled in the single-labeled sample.
The gold nanoparticles consist of approx. 85 atoms (size measurements, Figure S1 and S2). The number of atom can be calculated with the following formula (with *D* being the diameter of the particle):

$$N = n \cdot N_A = \frac{m}{M} \cdot N_A = \frac{\rho_{gold} \cdot \frac{1}{6}\pi D^3 \cdot N_A}{M}$$

with the density and atomic mass of gold $\rho_{gold} = 19{,}32\ \frac{g}{cm^3}$ and $M = 197\ \frac{g}{mol}$ one obtains:

$$N = 30.92\ (D[nm])^3$$

For simulations of their scattering pattern for different size gold particles (Figure S17), we modeled gold NP in an FCC lattice with the following radii and atom numbers:

$R = 7.04$ Å, $N = 87$
$R = 5.75$ Å, $N = 55$
$R = 4.98$ Å, $N = 43$
$R = 4.06$ Å, $N = 19$
$R = 2.87$ Å, $N = 13$

Here the radius is defined as the distance from the center Au-atom position to the outermost Au-atom position. Gold NP models are depicted in the inset in Figure S17.

Coarse-grained simulation of accessible volumes

For comparison to FPS and FRETraj distances we computed the accessible volumes also with a simple "sterics only" simulation custom written in python. The code reads in all atom positions and then creates a grid of possible label positions around the two attachment points, taken to be the C_β atoms of the cysteines introduced for labeling. The grid spacing is 1 Å. All positions that are further away from the attachment atoms than the linker length are deleted. Furthermore, all positions are deleted that are closer than the gold sphere radius (7 Å) to any atom in the protein. The remaining points form the accessible volume.

Supplementary Tables

	Subtraction: unlabeled single-labeled [%]	Distance, mean [Å]	Distance, uncertainty [Å]	Peak width, sigma [Å]	Peak width, uncertainty [Å]
**MalE$_{36-352}$**	**60-30**	56.2	0.3	1.9	0.1
	**30-20**	56.0	0.2	2.1	0.1
	**0-0**	55.8	0.2	2.2	0.1
**MalE$_{36-352}$ repeat**	**60-30**	56.2	0.2	2.0	0.2
	**30-20**	55.8	0.3	2.1	0.1
	**0-0**	55.9	0.1	2.1	0.1
**MalE$_{36-352}$ result**		**56.0**	**0.3**	**2.0**	**0.1**
**MalE$_{36-352}$ + 10 mM maltose**	**60-30**	43.3	0.7	3.3	1.0
	**30-20**	43.5	0.4	5.0	0.9
	**0-0**	42.7	0.6	6.6	1.5
**MalE$_{36-352}$ + 10 mM maltose repeat**	**60-30**	43.2	0.6	3.5	0.8
	**30-20**	43.1	0.5	4.9	0.9
	**0 - 0**	42.8	0.4	5.5	0.6
**MalE$_{36-352}$ + 10 mM maltose result**		**43.1**	**0.6**	**4.8**	**1.5**

Table S1. Measured AXSI distances for MalE$_{36-352}$. Distances are computed by fitting a Gaussian to the main peak of the *P*(d) distributions as illustrated in Figure S9 for 20 individual maximum entropy runs. The reported mean and uncertainty are the mean and standard deviation of 20 main peak positions from repeat runs of the maximum entropy code. Similarly, the width of the Gaussian peaks sigma is given with uncertainty. Both mutants with and without maltose were corrected for 60 % unlabeled and 30 % single-labeled, 30 % unlabeled and 20 % single-labeled or not corrected at all. The resulting distances are shown in the table. The result column shows the average for all subtractions and repeats. Values are discussed in the main text.

	Subtraction: unlabeled-single-labeled [%]	Distance, mean [Å]	Distance, uncertainty [Å]	Peak width, sigma [Å]	Peak width, uncertainty [Å]
**$MalE_{31-212}$**	**60-30**	63.3	0.2	2.9	0.2
	**30-20**	64.1	0.2	1.9	0.1
	**0-0**	63.7	0.6	1.9	0.1
**$MalE_{31-212}$ result**		**63.7**	**0.5**	**2.2**	**0.5**
**$MalE_{31-212}$ + 10 mM maltose**	**60-30**	59.3	0.4	2.8	0.4
	**30-20**	59.4	0.3	1.7	0.1
	**0-0**	59.1	0.2	1.8	0.1
**$MalE_{31-212}$ + 10 mM maltose repeat**	**60-30**	60.0	0.4	2.5	0.7
	**30-20**	59.3	0.2	1.7	0.1
	**0–0**	59.0	0.4	1.8	0.1
**$MalE_{31-212}$ + 10 mM maltose result**		**59.4**	**0.5**	**2.0**	**0.6**

Table S2. Measured AXSI distances for $MalE_{31-212}$. Equivalent to Table S1 but for $MalE_{31-212}$.

	**Linker length [Å]**	**Width [Å]**	**Radius 1 [Å]**	**Radius 2 [Å]**	**Radius 3 [Å]**
**Gold NP**	10	2	7	7	7
Alexa 555 – C2 Maleimide	21	4.5	8.8	4.2	1.5
Alexa Fluor 647 – C2 Maleimide	21	4.5	11	4.7	1.5

Table S3. Geometrical Parameters for the linkers used to model FRET and AXSI measurements. Values for Alexa fluorophores taken from[18].

Supplementary Figures

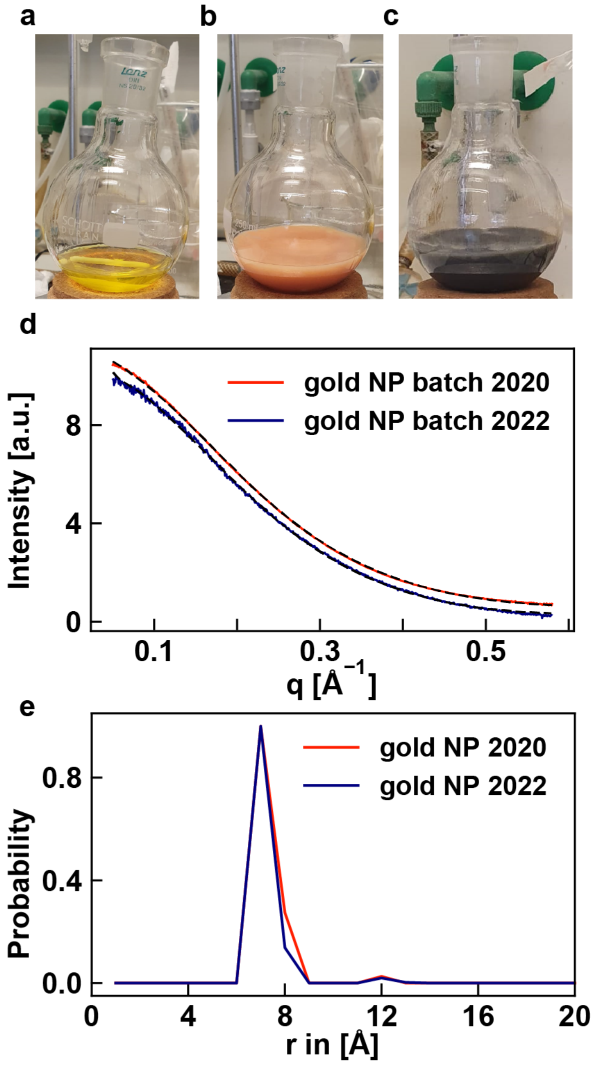

Figure S1. Preparation and SAXS characterization of gold nanoparticles for protein labeling. Gold solution during synthesis: **a)** Gold(III) chloride hydrate dissolved in 5:1 methanol:acetic acid. **b)** Gold(III) chloride hydrate solution after mixing with dissolved thioglucose. **c)** Same solution after dropwise addition of sodium borohydride. **d)** SAXS profiles for two independently synthesized batches of gold nanoparticles. SAXS data are shown in blue and red; black lines are fits of the profiles as a superposition of spheres with different radii. Data are vertically offset for clarity. **e)** Fitted distributions of sphere radii. The gold nanoparticles show good reproducibility across different synthesis runs and exhibit a narrow radius distributions with a mean of 7 Å.

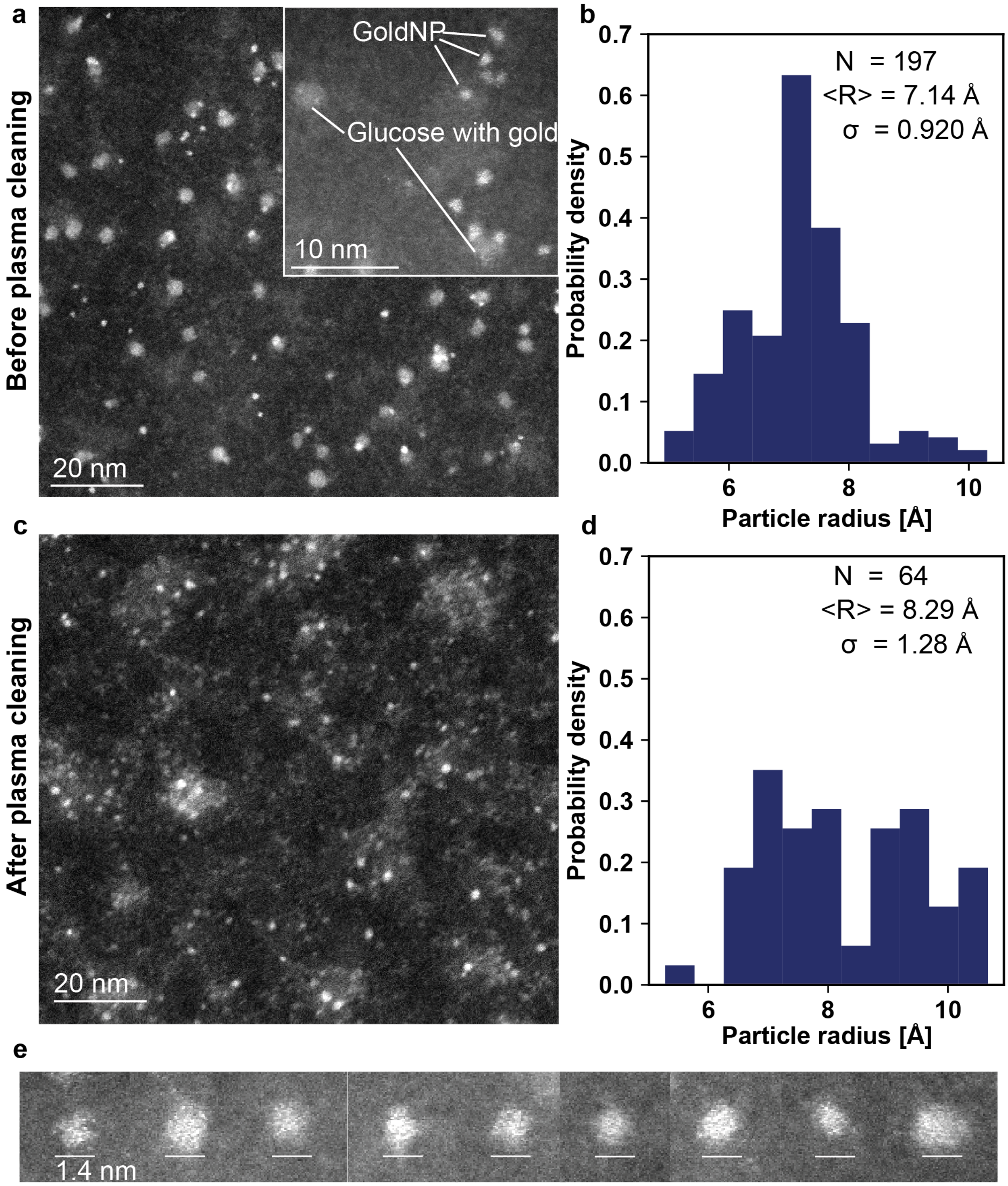

Figure S2: High-resolution TEM analysis of gold nanoparticles. Thioglucose passivated gold nanoparticles in ddH20 are dropcasted on a glow discharged functionalized Cu grid (QUANTIFOIL). TEM imaging used a Spectra 300 HAADF STEM probe 60 pm with a camera length of 145 mm. **a)** TEM images of the gold nanoparticle sample after first deposition. The image shows small, high-contrast nanoparticles in the expected size range. In addition, we observe larger particle contaminations with lower contrast. We hypothesize that the larger structures with lower contrast are formed by glucose with some gold content. **b)** Size distribution for the high contrast particles from images like the one shown in panel b). The mean radius is (7.14 ± 0.92) Å, which agrees well with the particle size distribution computed by SAXS (Figures S1). **c)** Same TEM grid after plasma cleaning in 5 % O_2 in Ar for 10 s. Plasma cleaning tends to removes carbon contaminations. The small, high-constrast

particles remain, albeit with somewhat more variable sizes. The larger particle contaminations disappear mostly, consistent with the hypothesis that lower contrast particles are predominantly carbon based. **d)** Size distributions of the high-contrast particles after plasma cleaning. The particle mean and standard deviation of the particle size distribution increased after plasma cleaning, likely due to sintering after (partial) removal of the stabilizing glucose shell. Particle size distributions in panels b) and d) were obtained by manually selecting and fitting circles to the particles. **e)** Gallery of high-resolution images of gold nanoparticles before plasma cleaning. All scale bars are 1.4 nm. The images reveal fairly round and monodisperse particles. The bright spots indicate the atomic sub-structure, but lattice parameters could not be determined, likely due important effects of the surface for these small particles[19].

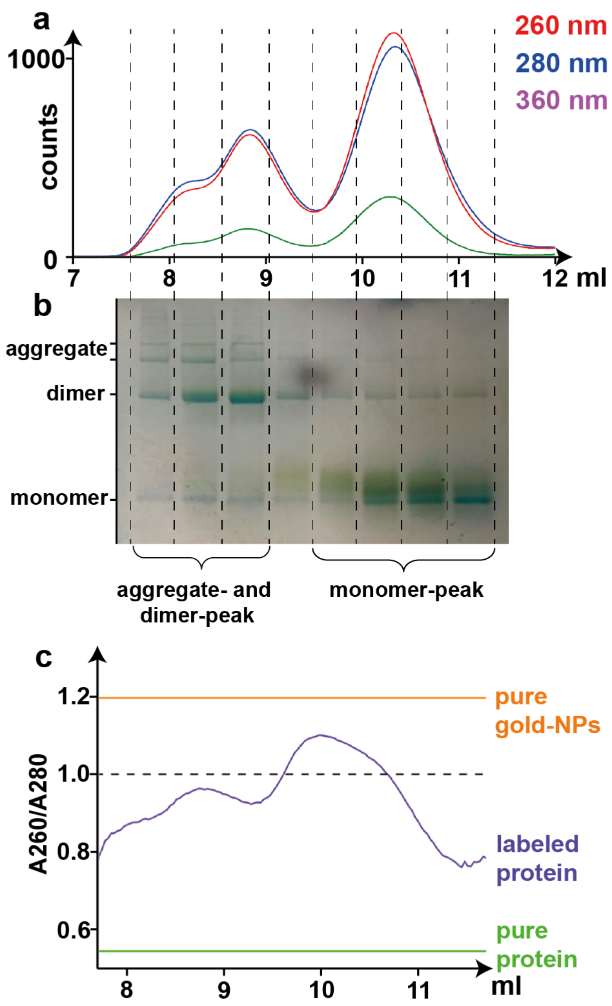

Figure S3. Protein labeling and purification. **a)** HPLC elution profile with a Superdex® Increase 75 column and **b)** SDS page gel (Mini-Protean TGX® PAGE gel any kD, Bio-Rad®) electrophoresis (denatured) of gold-labeled MalE36-352. The first peak with a shoulder on the left from 7 to 9 ml corresponds to mostly aggregates and dimers with a lower fraction of gold. The right peak (between 9.5 and 11 ml) consists of mostly monomeric proteins. The gel was stained with Coomassie blue and LI Silver enhancement (greenish), which stains the gold NPs. The lanes in the gel correspond to fractions in the HPLC run (indicated by dashed lines). The upper two bands correspond to protein clusters with MW > 113 kDa. The third band corresponds to dimers with MW of 81-83 kDa. The lower triple band corresponds to protein monomers with MW from 41-53 kDa. We note, that molecular weight estimates are not accurate for gold NPs. **c)** Ratio of 260 nm to 280 nm absorbance. The ratio is above 1 for the left flank of the monomer peak. For ASAXS measurements only the left flank of the monomer-peak was selected. From the HPLC run we can also compute the protein:gold concentration ratio in the selected fractions. For the run shown here we get a ratio of 1:0.75 (see Figure S3 caption for calculation). However, small gold and protein concentrations were used for this preparation. Labeling high concentrations of proteins, which is necessary for SAXS measurements, reduces the protein:gold ratio to ~1:0.5 (high concentration HPLC runs, shown in Figure S4).

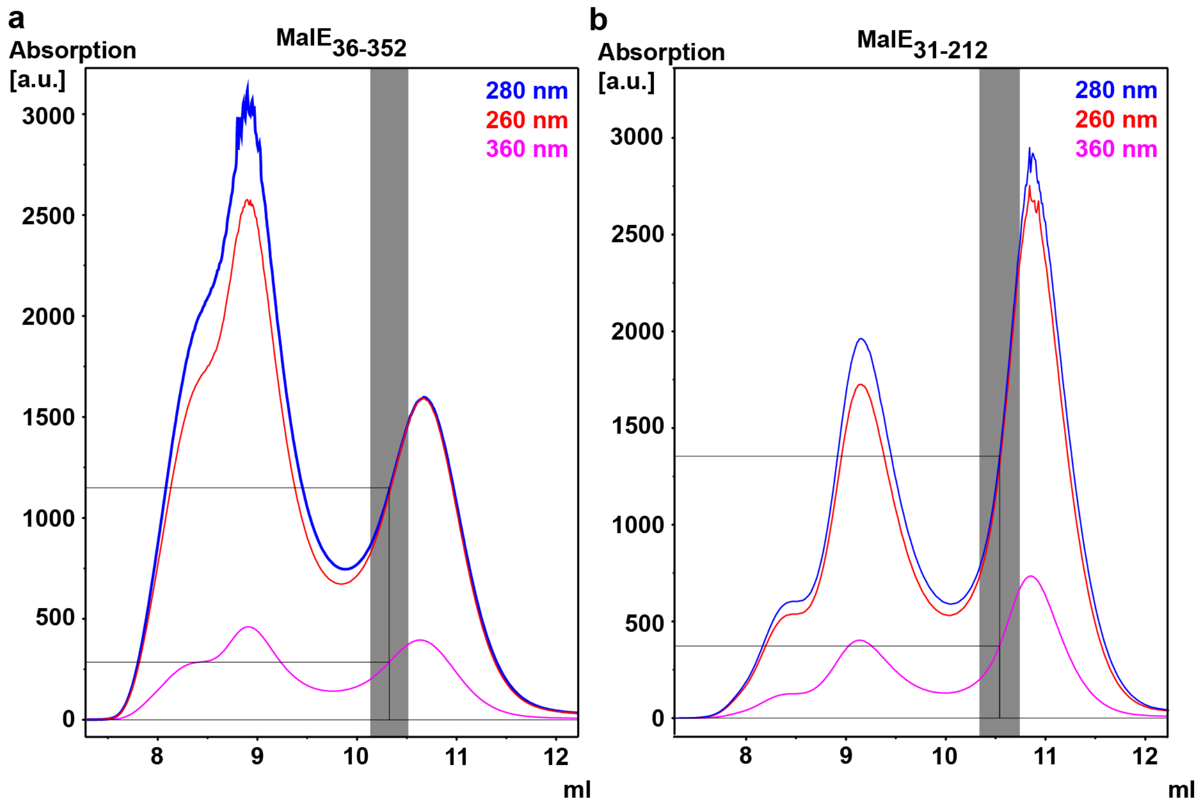

Figure S4. Size exclusion chromatography runs for larger amounts of MalE$_{31\text{-}212}$ and MalE$_{36\text{-}352}$. Absorption was measured at 260, 280 and 360 nm. Grey backgrounds indicate the fractions that were used for AXSI measurements. The extinction coefficient of gold NPs[9] is $\varepsilon = 7.6 \cdot 10^4 \frac{1}{Mol \cdot cm}$ at 360 nm. The gold absorption at 280 nm is $\varepsilon = 15.85 \cdot 10^4 \frac{1}{Mol \cdot cm}$ and the proteins used here do not absorb at 360 nm and have an extinction coefficient of $\varepsilon = 6.65 \cdot 10^4 \frac{1}{Mol \cdot cm}$ at 280 nm (computation[20]). To compute the protein:gold ratio we used the counts in the center of the selected fractions (see vertical and horizontal black lines). At 360 nm all counts originate from gold NPs. We can calculate the absorption that originates from gold NPs at 280 nm with the following formula: $counts_{gold,280\,nm} = counts_{gold,360\,nm} \cdot \frac{\varepsilon_{gold,280\,nm}}{\varepsilon_{gold,360\,nm}}$. The protein absorption at 280 nm is then given by: $counts_{prot,280\,nm} = counts_{total,280\,nm} - counts_{gold,280\,nm}$. Concentrations are given by the counts divided by the extinction coefficients. For MalE$_{36\text{-}352}$ we obtain a protein:gold ratio of 1:0.45 and for MalE$_{31\text{-}212}$ we obtain 1:0.56. We note that the protein:gold ratio for a perfectly double-labeled sample is 1:2. The measured ratios ~1:0.5 agree with our estimate of 30% single-labeled and 10% double-labeled sample from the scattering profiles.

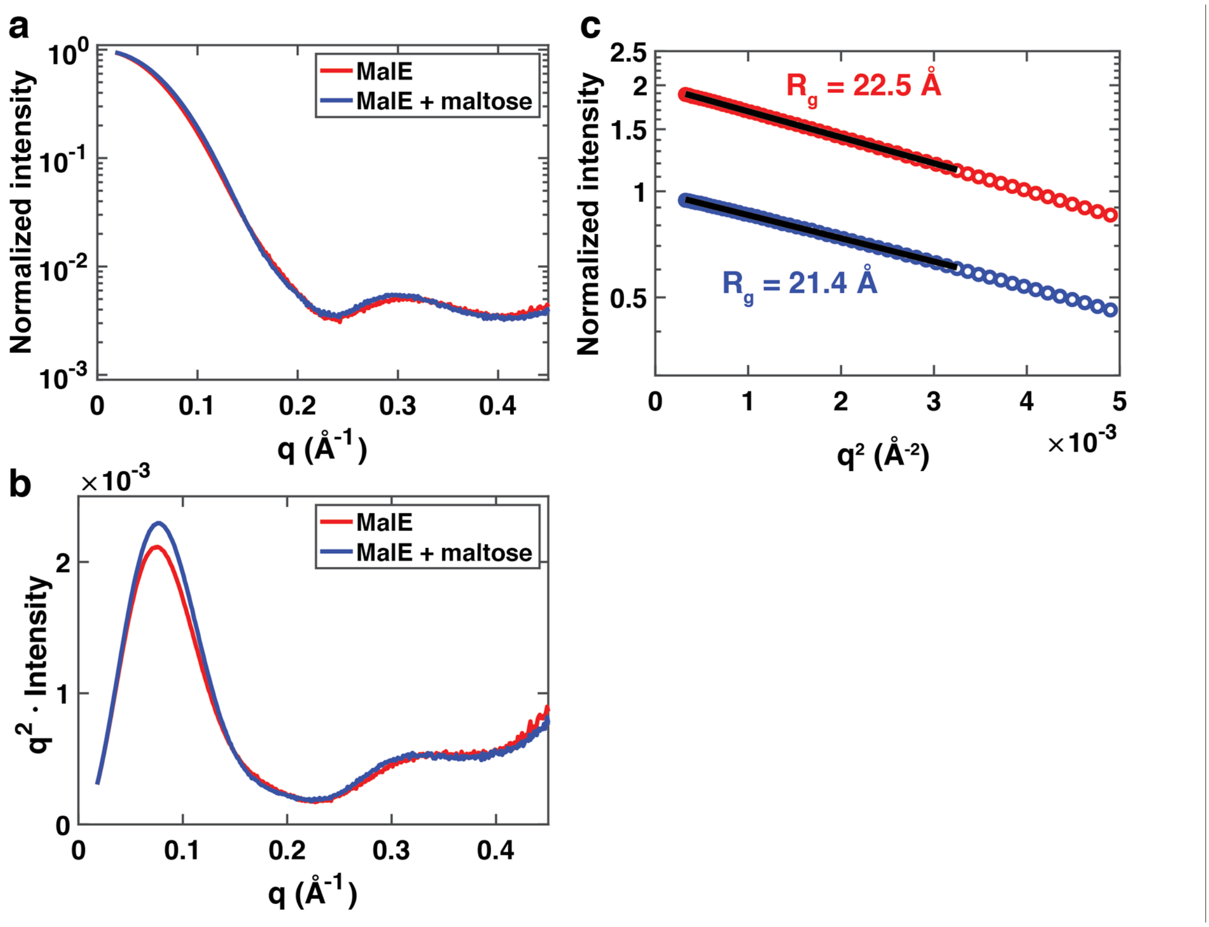

Figure S5. SAXS analysis for unlabeled MalE in the apo and holo configuration. Normalized SAXS data for $MalE_{36\text{-}352}$ without gold labels at a fixed energy of 11.619 keV. **a)** Normalized scattering profiles of $MalE_{36\text{-}352}$ in the absence of maltose (red) and in the presence of 10 mM maltose (blue). The profiles show subtle differences, but are overall similar. **b)** Kratky representation (scattering intensity weighted by q^2 vs. q) of the same data as shown panel a. The Kratky representation highlights the conformational change upon addition of maltose, as has been observed for similar bi-lobed proteins previously[21, 22]. **c)** Guinier analysis of the scattering profiles shown in panel a. The logarithm of the scattering intensity vs. q^2 for the lowest q values is well described by a straight line (shown in black), indicative of a dilute and monodisperse sample. From the slope of the linear fits, we determine the radius of gyration to be R_g = 22.5 Å ± 0.3 Å in the absence of maltose and R_g = 21.4 Å ± 0.3 Å in the presence of 10 mM maltose. Errors are from the uncertainty of the fits. The data set in the absence of maltose (red) is scaled by a factor of 2 for clarity. The experimentally determined radii of gyration are in excellent agreement with previous experimental results[21] and with the values computed from the crystal structures of the open[23] (PDB accession code 1OMP; Rg = 22.8 Å; radii of gyration were computed using CRYSOL[17] with default settings) and closed, maltose bound conformation[24] of MalE (PDB accession code 1ANF; Rg = 21.8 Å).

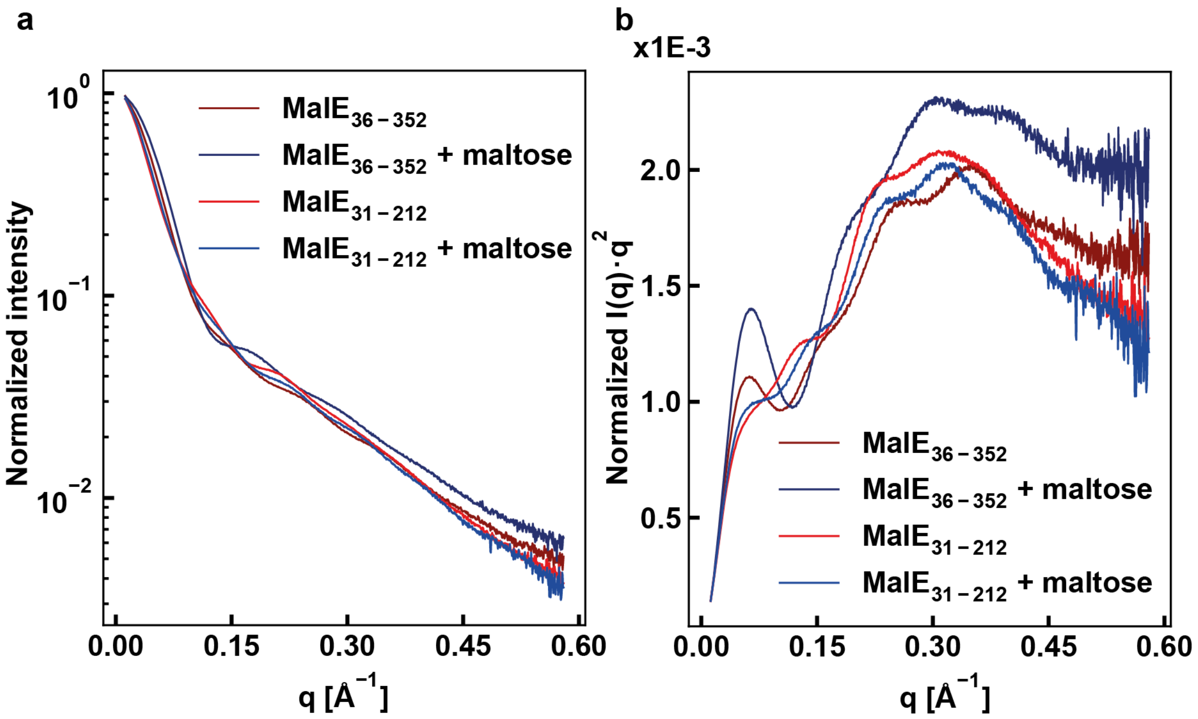

Figure S6. Scattering profiles for gold-labeled MalE for MalE$_{36\text{-}352}$ and MalE$_{31\text{-}212}$ with and without maltose. Data are an alternative representation of the data in Figure 2. The profiles shown are the "off-edge" (at −300 eV) data from Figure 2a. **a)** Normalized scattering intensity vs. q. **b)** Scattering data in Kratky representation of (normalized) scattering intensity weighted by q^2. All four scattering profiles show oscillations due to the gold labels. At the same time, all four conditions give rise to clearly distinct scattering patterns.

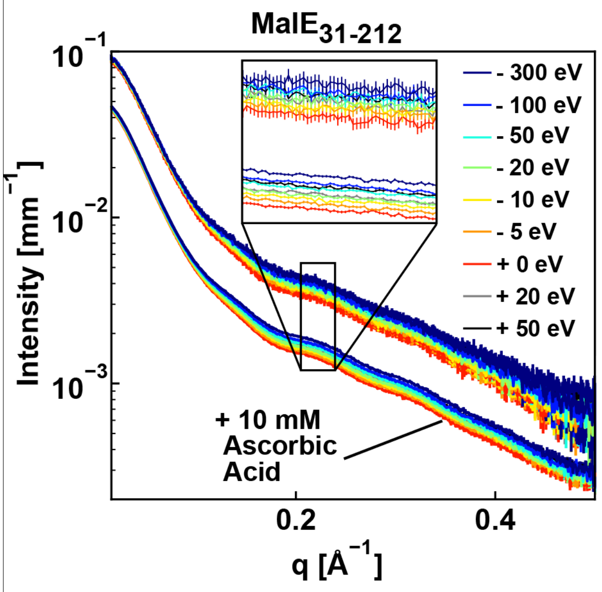

Figure S7. Effect of exposure time and averaging on the signal-to-noise ratio. ASAXS data for double-labeled $MalE_{31\text{-}212}$. Upper curves are the average of 5 frames with 0.3 s exposure time each and the photon flux was $2.3 \cdot 10^{11}$ photons/s. The lower curves are the average of 8 frames with 0.5 s each and a flux of $7.3 \cdot 10^{12}$ photons/s. The higher flux and longer exposure time are made possible by addition of 10 mM ascorbic acid. The sample in the capillary was moved between frames. Radiation damage was not observed.

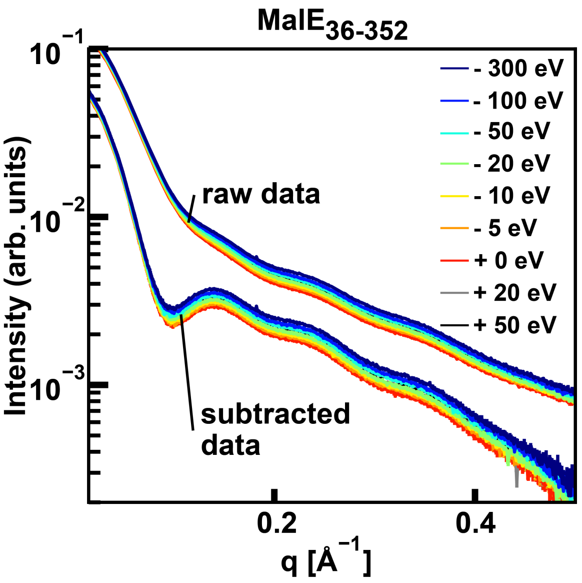

Figure S8. Scattering Data after the subtraction of unlabeled and single-labeled proteins. Similar to the buffer subtraction we subtracted 60% of unlabeled and 30% of single-labeled MalE proteins from the measurements that include the double-labeled sample. Matching single-labeled and unlabeled proteins were also measured at the same energies.

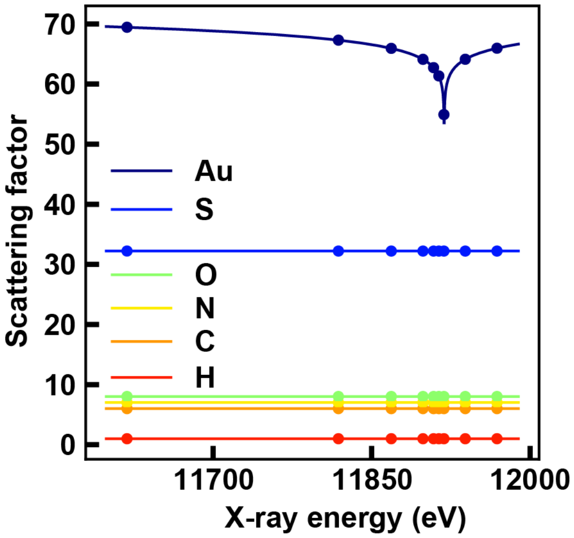

Figure S9. Real part of atomic scattering factors. Atomic scattering factors for all atoms present in the sample. Points depict the 9 energies selected for AXSI measurements. The scattering factors for the atoms present in the protein show almost no energy dependence in the chosen energy regime, while the gold LIII-absorption edge of gold is clearly visible as a minimum at 11919 eV.

Data taken from: http://skuld.bmsc.washington.edu/scatter/AS_periodic.html

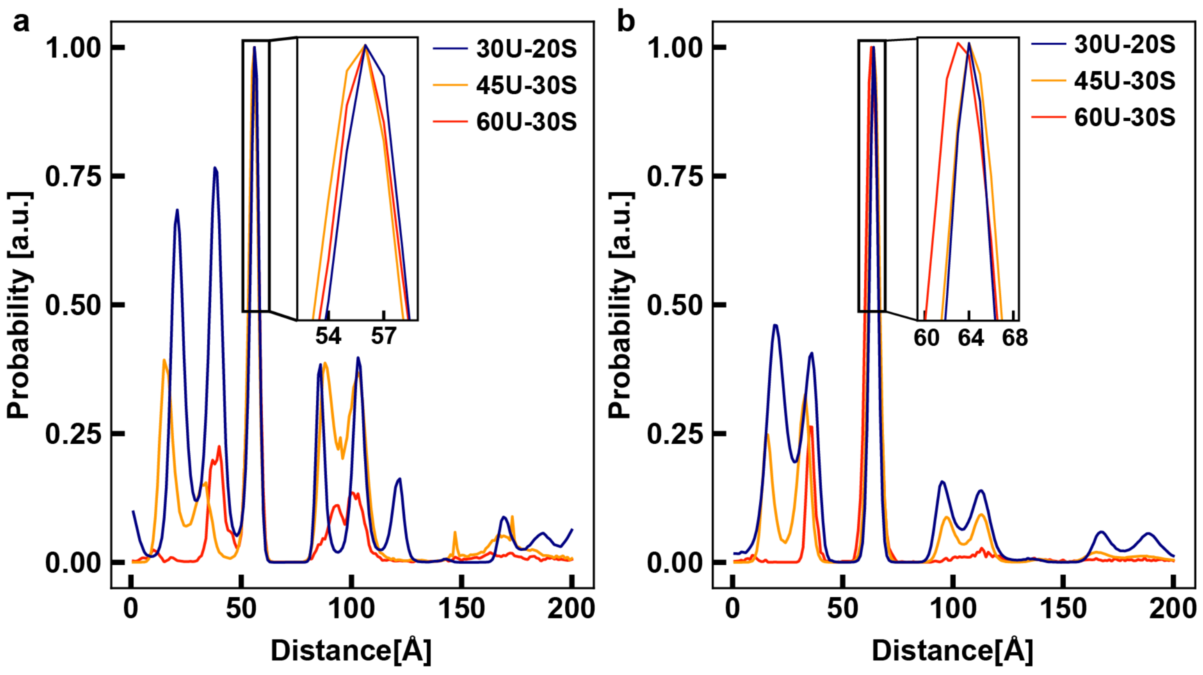

Figure S10. Distance distributions for both MalE variants for different background subtraction schemes. Data are for **a)** MalE$_{36\text{-}352}$. **b)** MalE$_{31\text{-}212}$. Example distributions for different subtraction of unlabeled and single-labeled. 30% unlabeled 20% single-labeled (blue), 45% unlabeled 30% single-labeled (orange) and 60% unlabeled 30% single-labeled (red). The zoom in depicts the main peak positions.

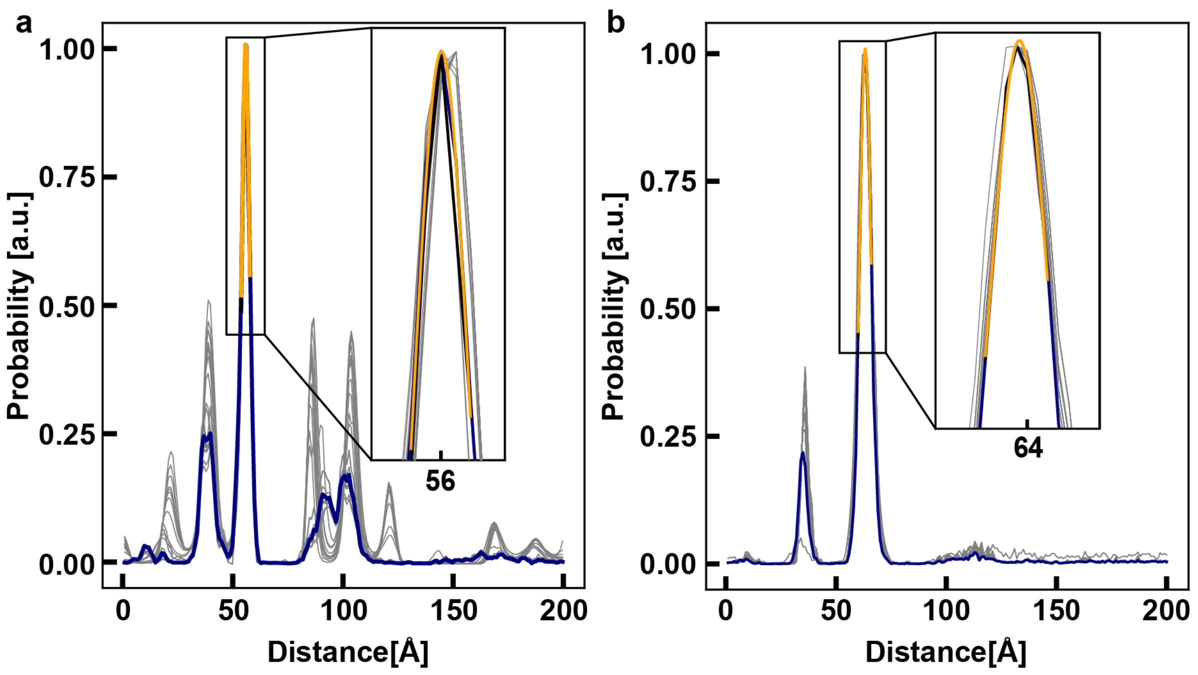

Figure S11. Distance distributions from repeat runs of the maximum entropy inversion algorithm. Data are for **a)** $MalE_{36-352}$ and **b)** $MalE_{31-212}$. 20 distance distributions obtained from repeat runs of the maximum entropy inversion scheme using the scattering data after subtraction of 60% unlabeled and 30% double-labeled are shown (grey thin lines). The mean peak position is given in Tables S1 and S2. One distribution is shown in blue. The Gaussian fit that is used to determine the peak center and standard deviation is shown in orange. The inset shows a zoom on the main peak position.

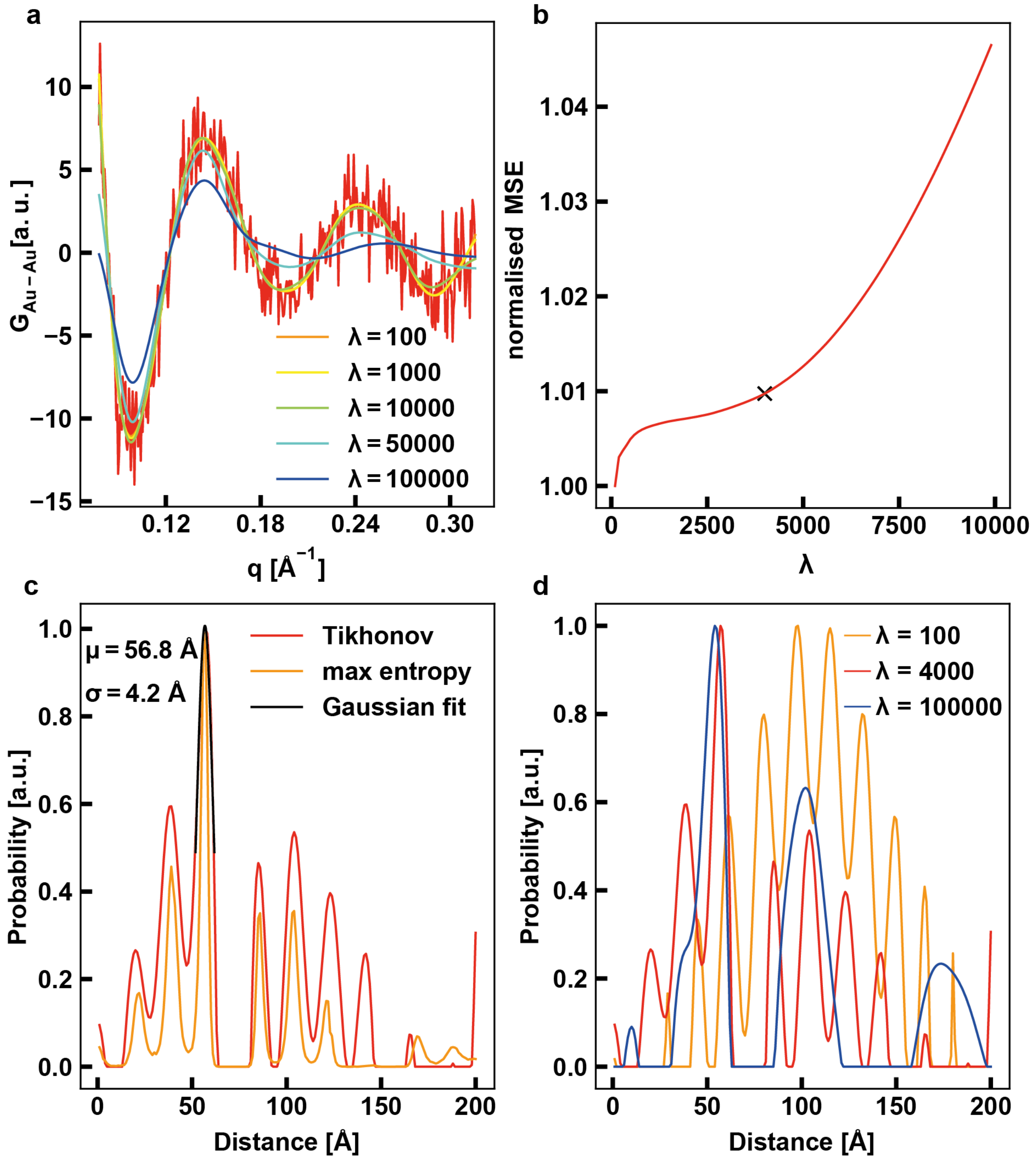

Figure S12: Tikhonov regularization as an alternative to maximum entropy to compute *P*(d) distributions from the scattering interference terms for MalE36-352. a) Experimental gold-gold interference term G_{Au-Au} for MalE36-352 apo (red data) together with fits using Tikhonov regularization for different values of the regularization parameter λ. We want to solve $IqD \cdot P(d) = G_{Au-Au}$. Where IqD are the basis functions for a pair of gold spheres with distance d and $P(d)$ is the probability of a given distance. For Tikhonov regularization we construct a Matrix: $IqD_\lambda = \begin{bmatrix} IqD \\ \lambda I \end{bmatrix}$ where I is the identity matrix and the vector $g = \begin{bmatrix} G_{Au-Au} \\ 0 \end{bmatrix}$. We then use scipy's NonNegative Least Squares function and minimize $||IqD_\lambda \cdot P(d) - g||^2$ to obtain P(d). **b)** Mean squared error for the fits to the gold-gold interference as a function of the regularization parameter λ. λ should be as large as possible without losing much fitting accuracy. We chose a λ of 4000 marked as a black cross on the curve. **c)**

Distance distribution for λ = 4000 (red) compared to the distance distribution of the maximum entropy algorithm (orange). We note that the position of the main peak is not changed, but that the standard deviation of Gaussian fitted to the main peak is increased (roughly doubled in this case) for Tikhonov regularization (σ = 4.2 Å) compared to maximum entropy (σ = 2.0 Å). **d)** Example distributions for λ = 100, 4000 and 100 000. λ = 100 (orange) is an example for overfitting with a good fit to G_{Au-Au}, however the distribution consists of too many peaks and no main peak. λ = 4000 (red) gives a distribution with fewer peaks and a single main peak. λ = 100 000 (blue) is an example for oversmoothing with even fewer peaks, but the main peak starts to shift and G_{Au-Au} is not adequately fitted anymore (blue data in a).

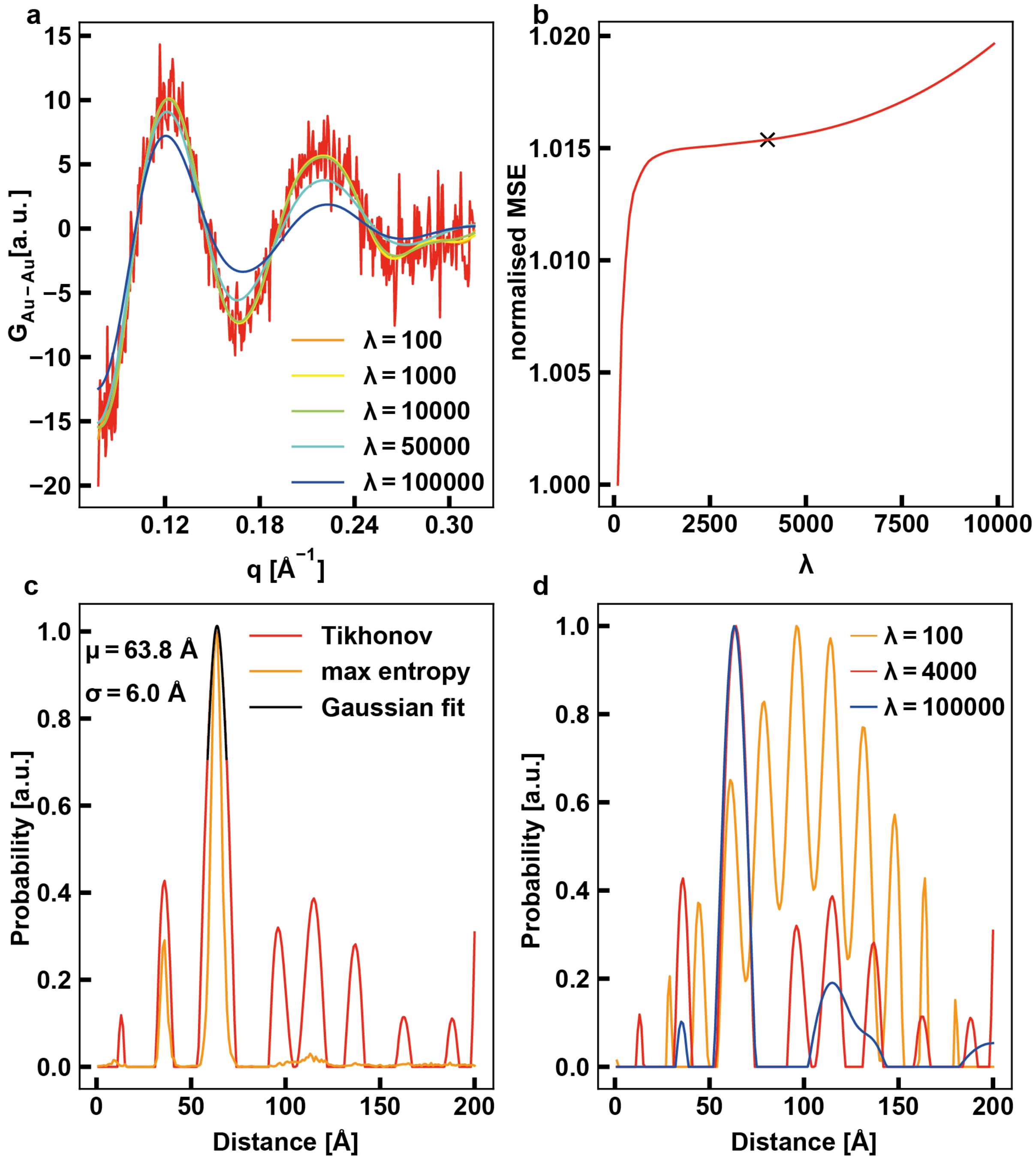

Figure S13: Tikhonov regularization as an alternative to maximum entropy to compute *P*(d) distributions from the scattering interference terms for MalE31-212. Panels are similar to Figure S12. **a)** Experimental gold-gold interference term G_{Au-Au} for MalE31-212 apo (red data) together with fits using Tikhonov regularization for different values of the regularization parameter λ. **b)** Mean squared error for the fits to the gold-gold interference as a function of the regularization parameter λ. We chose a λ of 4000 marked as a black cross on the curve. **c)** Distance distribution for λ = 4000 (red) compared to the distance distribution of the maximum entropy algorithm (orange). We note that the position of the main peak is not changed, but that the standard deviation of Gaussian fitted to the main peak is increased (roughly tripled in this case) for Tikhonov regularization (σ = 6.0 Å) compared to maximum entropy (σ = 2.2 Å). **d)** Example distributions for λ = 100, 4000 and 100 000.

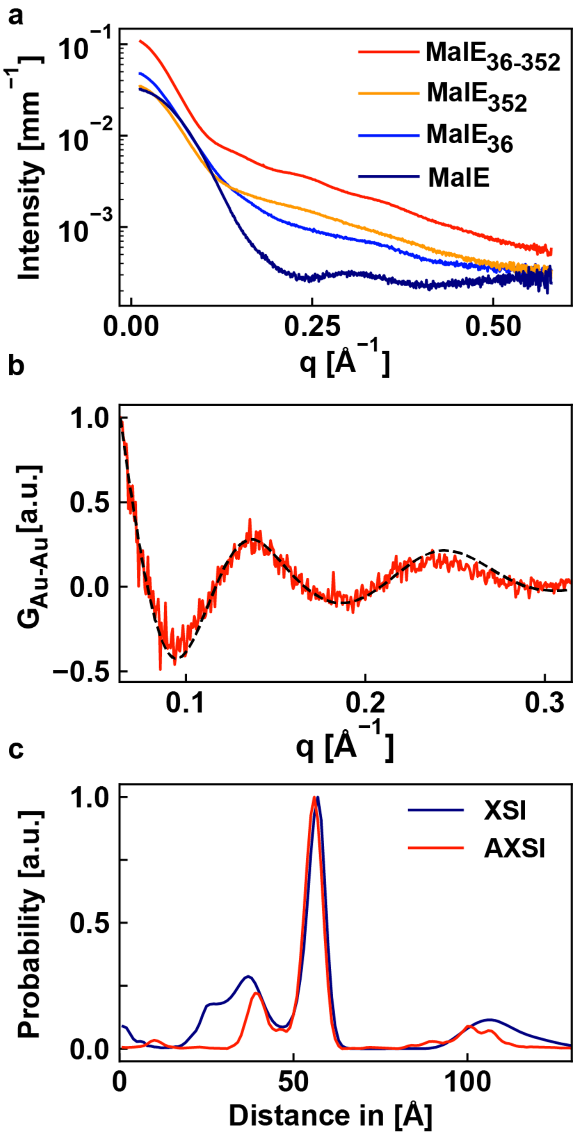

Figure S14. Distance distribution for MalE$_{36\text{-}352}$ by single energy XSI. a) SAXS measurements on MalE36-352 with two labels (I_{double}), one label MalE36 ($I_{single,1}$) at amino acid position 36, one label MalE352 at 352 ($I_{single,2}$) and unlabeled MalE ($I_{unlabeled}$) measured at 11.619 keV. b) Interference term $G_{Au-Au} = I_{double} - \left(I_{single,1} + I_{single,2} - I_{unlabeled}\right)$ calculated with the regular XSI method (red line) and the fit (black dashed line). Details of the regular XSI procedure in[25] c) Comparison of the distance distributions computed with AXSI for MalE$_{36\text{-}352}$, where we subtracted 60% single-labeled and 30% unlabeled protein, and the single energy XSI method.

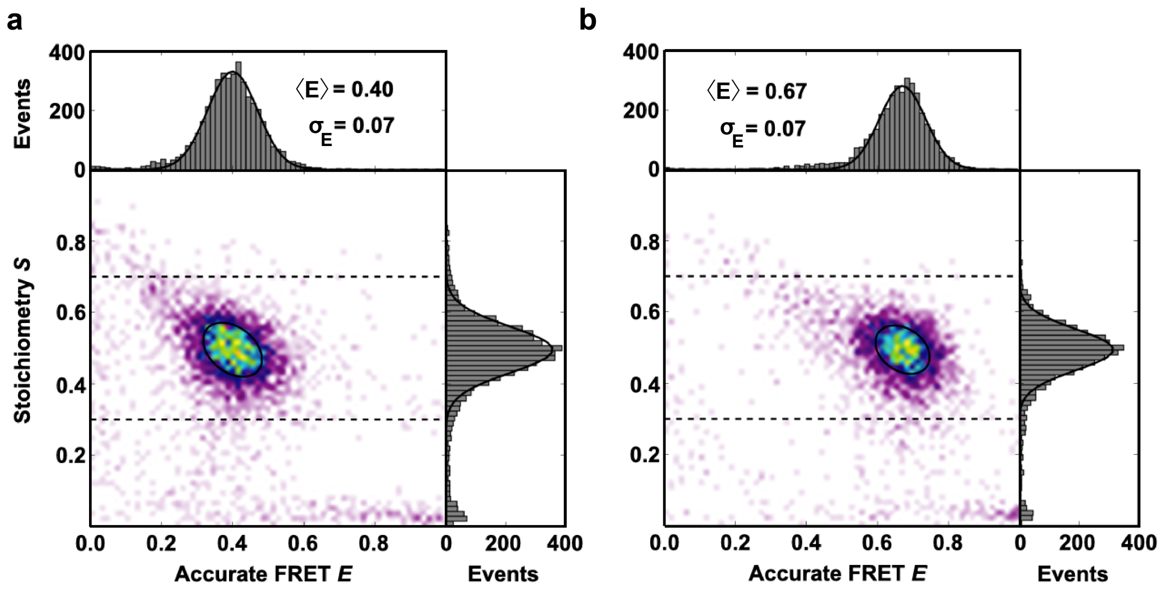

Figure S15. Monitoring conformational changes in MalE36-352 by smFRET/ALEX. Corrected accurate *ES*-histograms of $MalE_{36\text{-}352}$ labeled with Alexa555 and Alexa647 for the open conformation in the absence of maltose (a) and the liganded, closed (b) conformation in the presence of 1 mM maltose.

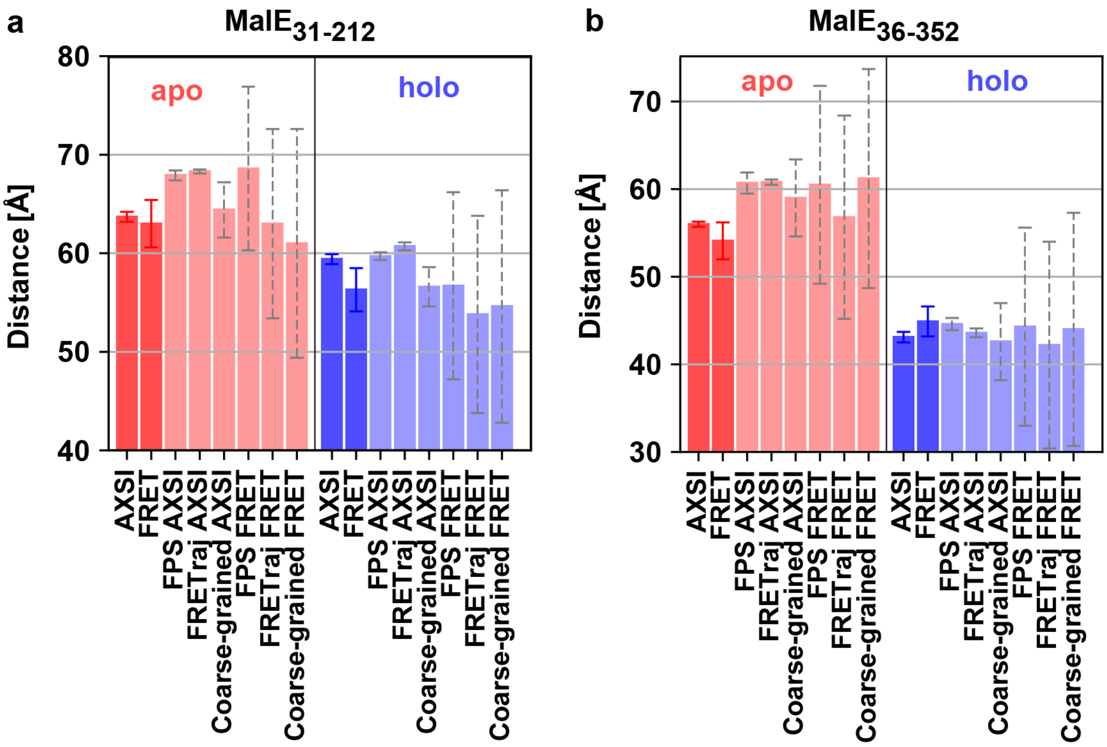

Figure S16. Visualization of measured and computed distances between AXSI and FRET labels for different MalE constructs. The open/apo state is depicted in red and the closed/holo state in blue. AXSI and FRET data shown in darker, simulation data in lighter blue/red. Error bars for the experiment data are experimental errors of the mean. Simulation errorbars depict the standard deviations of all possible distances between the accessible volumes (AVs) or accessible contact volumes (ACVs).

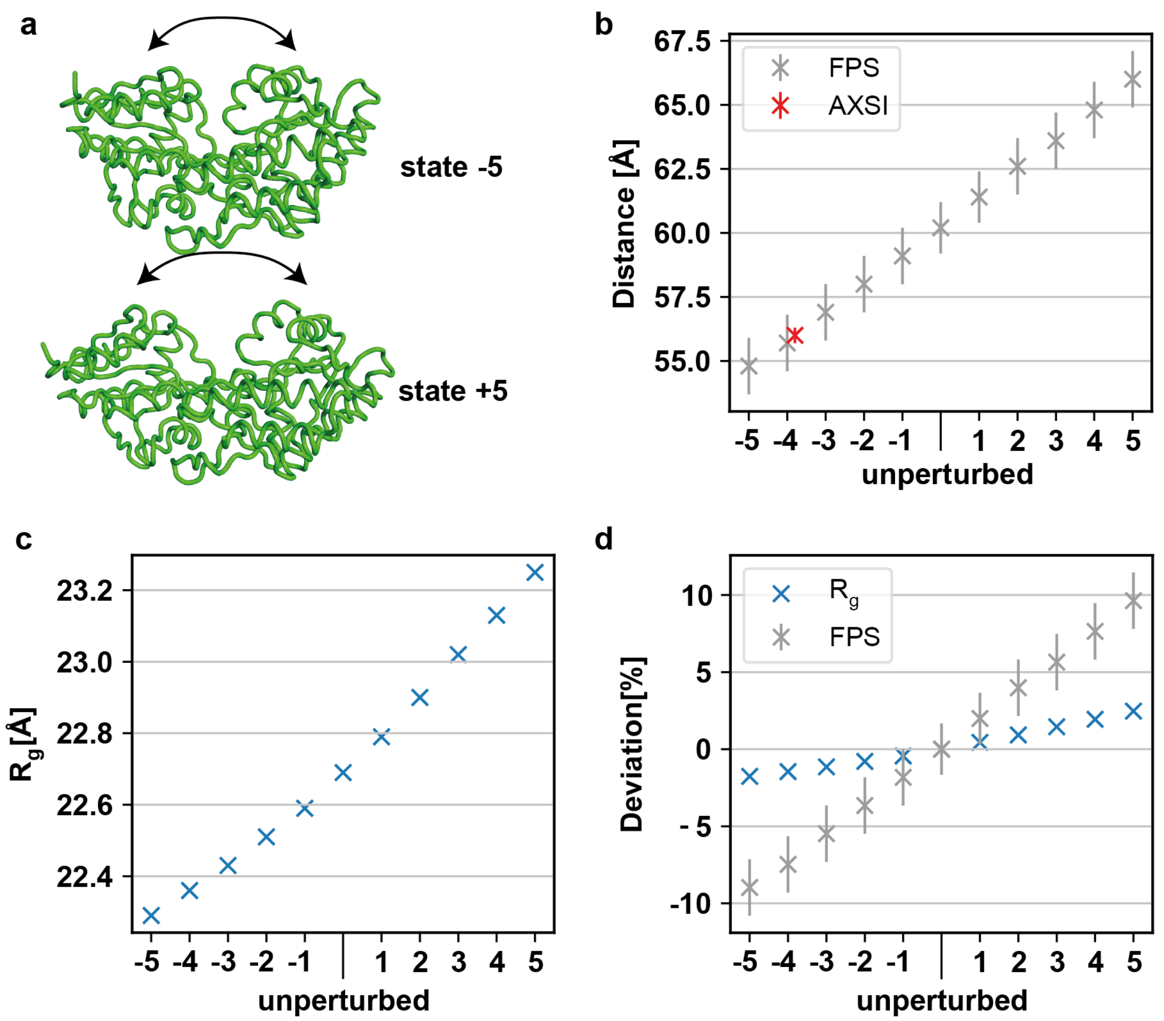

Figure S17. Elastic network simulation for MalE$_{36\text{-}352}$ in the apo state. **a)** Illustration of protein movement computed by normal model tool elNemo[26] along the first non-trivial normal mode (mode 7) using PDB 1OMP as an input. **b)** FPS distances for a 10 Å long linker and 7 Å radius gold NPs for the motion along the normal mode 7. The unperturbed state corresponds to the crystal structure. The numbers on the x-axis correspond to configurations obtained by perturbing the structure along mode 7 by $dq = -100$ to 100 in steps of 20. In red the AXSI measurement for MalE$_{36\text{-}352}$ is shown. **c)** R_g-values for the same protein conformations computed with CRYSOL. **d)** Percentage deviation of the R_g and the residue 36 to 352 distance from the unperturbed state. We note that a similar deformation along the first non-trivial normal mode improves the agreement between experimental and predicted values both for the mean distance between labels at residues 36 and 352 and the radius of gyration (the experimentally determined R_g is 22.5 Å ± 0.3 Å; Figure S5). However, the change for the R_g is within experimental error. In general, the relative changes in R_g are much smaller than for the label-label distances for this protein system, suggesting that the label-label distance is a better experimental observable.

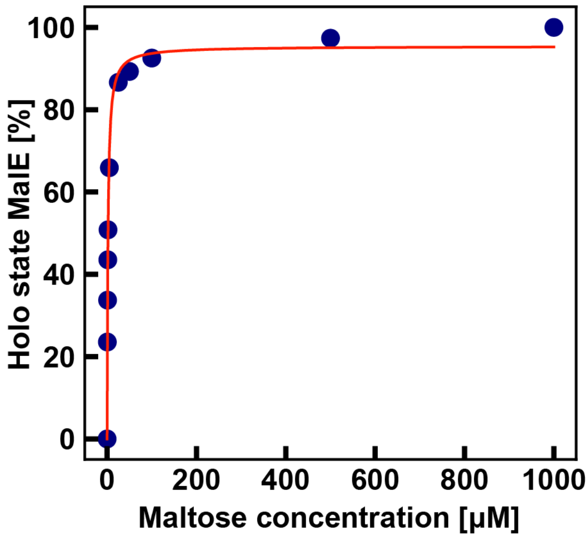

Figure S18. Maltose titration of maltose binding protein variant MalE$_{31\text{-}212}$ with FRET labels. We performed FRET measurements similar to the ones shown in Figure 3 on MalE$_{31\text{-}212}$ labeled with AlexaFluor555 and AlexaFluor647. The maltose binding curve was calculated based on the smFRET measurements considering the closed fraction as a function of maltose concentration. The open and closed fractions per measurement condition (blue circles) were identified by a two-state Gaussian fit with fixed values for the apparent FRET efficiency (E, σ_E), the Stoichiometry (S, σ_S) and the two-dimensional shape of the population. Population amplitude values for E were weighted with the respective matching σ_E and a simple binding isotherm with one single binding site was used to fit the affinity of labeled MalE$_{31\text{-}212}$ towards maltose (red curve). The parameters for each population were obtained from individual ES-FRET histograms of ligand-free open- and saturated closed-state (1 mM maltose) MalE, respectively. The following formula was used for the fit: $c_{holo}[\%] = c_{holo,max}[\%] \cdot c_{maltose}[\mu\text{M}]/(K_d[\mu\text{M}] + c_{maltose}[\mu\text{M}])$. We obtain the following fit parameters: $K_d = (1.84 \pm 0.13)$ µM and $c_{holo,max} = (95.4 \pm 1.3)\%$ with $R^2 = 0.994$. The K_d for the second mutant MalE$_{36\text{-}352}$ towards maltose was previously determined to be $(2.2 \pm 0.4)\ \mu M$ using microscale thermophoresis[18], in agreement, within experimental error with our results for MalE$_{31\text{-}212}$. These finding are consistent with previous work that has shown that similar MalE mutants have affinities that are very close and essentially within experimental error of one another, irrespective of whether the protein is labeled or unlabeled, and also within experimental error of the wild type protein[2, 7, 24, 27, 28].

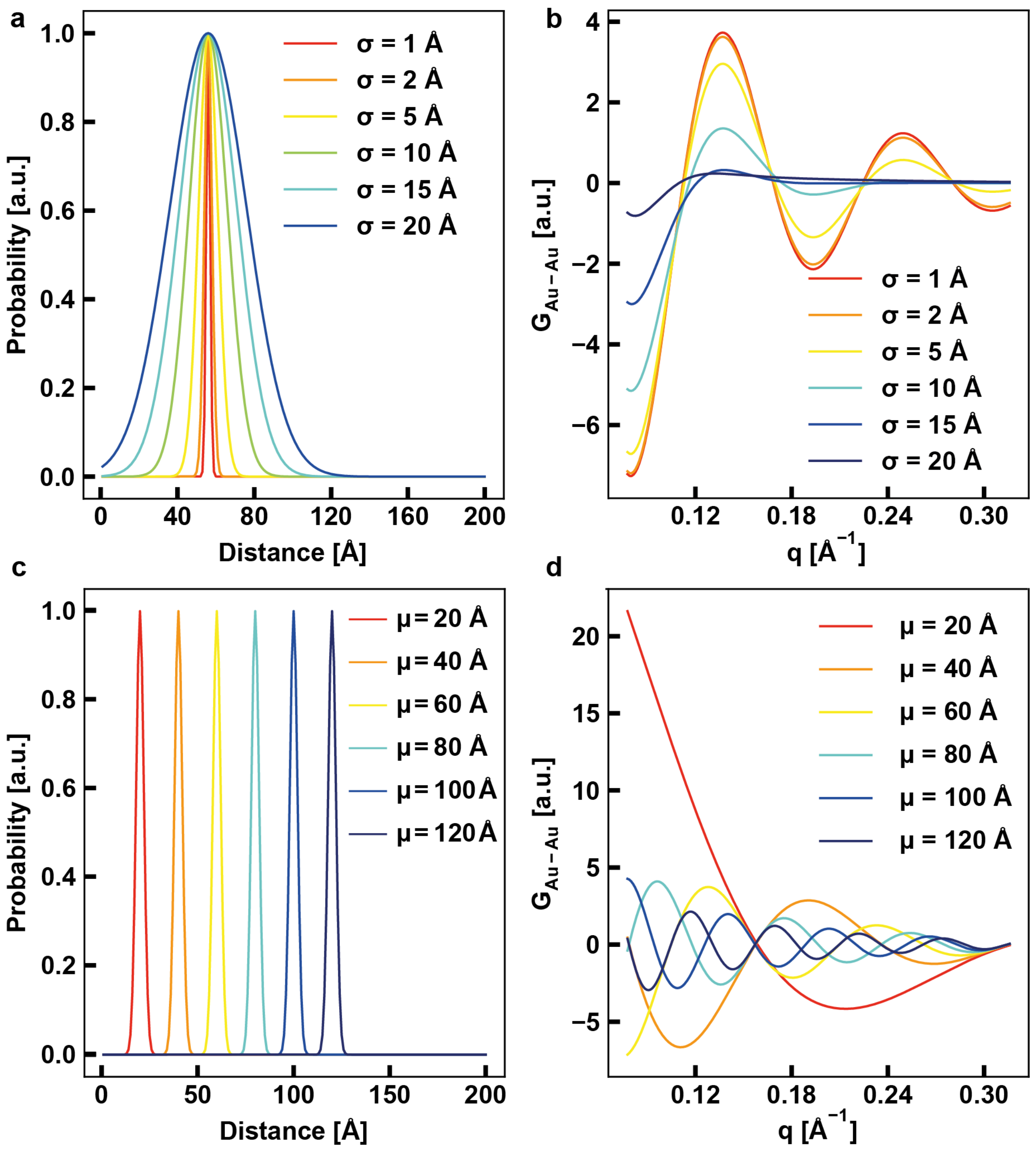

Figure S19. Effects of different peak widths and peak positions on the scattering interference terms. We simulated different idealized label-label distance distributions *P*(d) as Gaussian with mean μ and standard deviation σ. We then computed the corresponding scattering interference terms to explore the effects of the width and position of the distance distributions. To compute the scattering interference from the distance distribution, a set of basis functions for gold spheres with the size distribution that we measured experimentally was computed. The basis functions[9] are the scattering of a pair of gold spheres with distances from 1 Å to 200 Å. Multiplying the basis functions with the probability of certain distances and summing over all distances gives the interference. **a)** *P*(d) with peaks positioned at 56 Å (the distance measured for $MalE_{36\text{-}352}$ apo) with different peak widths σ. **b)** Gold-gold interference terms for normalized *P*(d) in panel a). **c)** *P*(d) with peaks at different distances μ all with σ = 2 Å. **d)** Interference terms corresponding to the data in panel c.

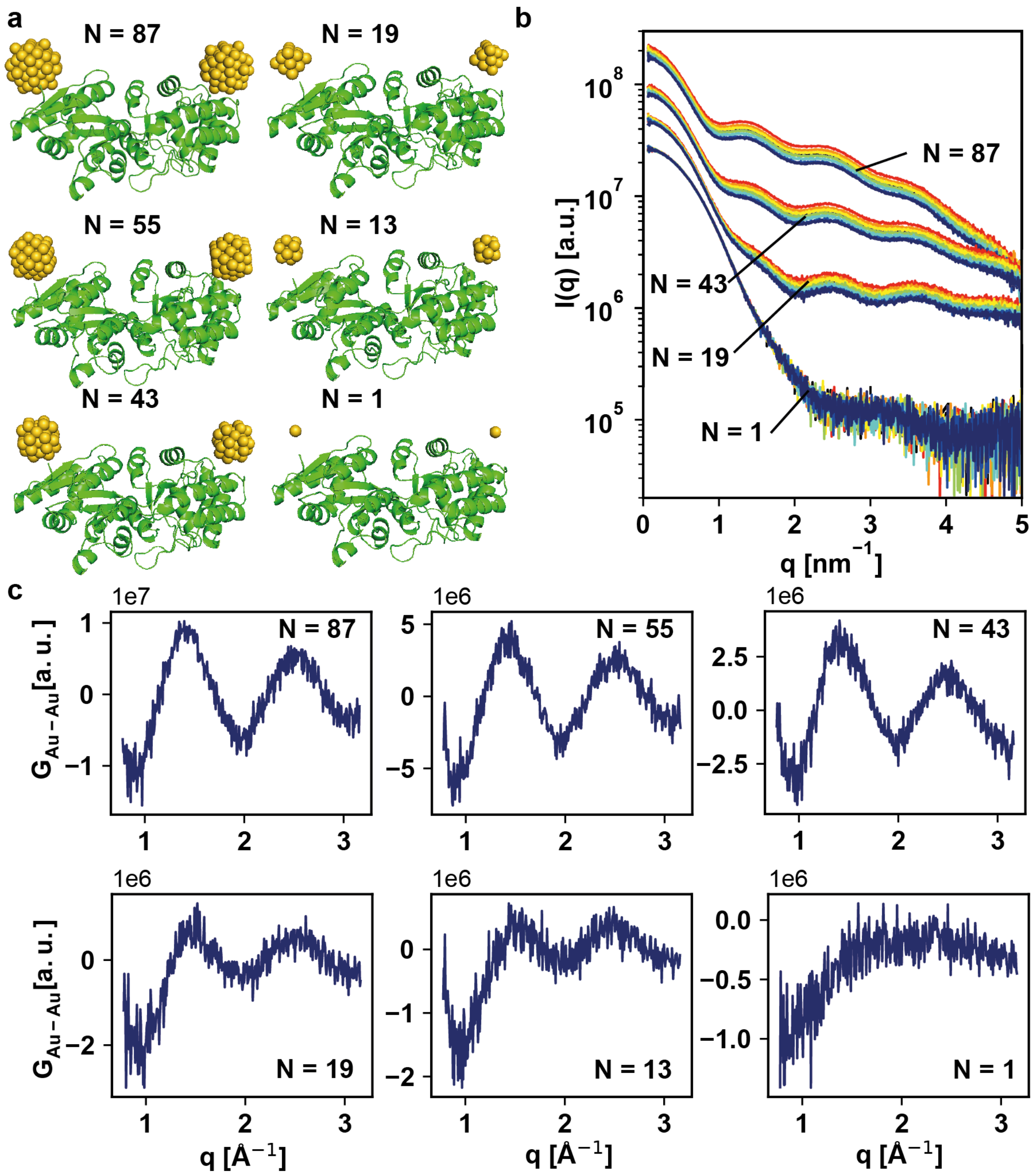

Figure S20. Simulation of AXSI data for different gold nanoparticle sizes to investigate resolution. Simulations were carried out in CRYSOL for $MalE_{36\text{-}352}$ in the apo conformation. a) Visualizations of $MalE_{36\text{-}352}$ with gold NPs with different sizes. N=87 corresponds to the approximate particle size used in our experiments. The gold NPs were positioned in the computed accessible volume (coarse-grained method) with a distance of 56 Å (experimentally measured value). b) Scattering curves for double-labeled $MalE_{36\text{-}352}$ simulated with CRYSOL for different N. We added Gaussian noise with the same standard deviation that we observe for $MalE_{36\text{-}352}$ apo after subtraction of the single and unlabeled contributions. c) Resulting interference terms for different numbers of gold atoms. To obtain clearly visible oscillations, gold particles with approximately $N = 40$ atoms are required at the current level of signal-to-noise and it would likely be impossible to obtain reliable distance distribution from single atoms as labels.

Supplementary References

1. M. F. Peter, C. Gebhardt, R. Mächtel, G. G. M. Muñoz, J. Glaenzer, A. Narducci, G. H. Thomas, T. Cordes and G. Hagelueken, *Nature Communications*, 2022, **13**, 4396.
2. M. de Boer, G. Gouridis, R. Vietrov, S. L. Begg, G. K. Schuurman-Wolters, F. Husada, N. Eleftheriadis, B. Poolman, C. A. McDevitt and T. Cordes, *Elife*, 2019, **8**, e44652.
3. C. Ganesh, F. N. Zaidi, J. B. Udgaonkar and R. Varadarajan, *Protein Sci*, 2001, **10**, 1635-1644.
4. G. Gouridis, G. K. Schuurman-Wolters, E. Ploetz, F. Husada, R. Vietrov, M. De Boer, T. Cordes and B. Poolman, *Nature Structural & Molecular Biology*, 2015, **22**, 57-64.
5. A. N. Kapanidis, N. K. Lee, T. A. Laurence, S. Doose, E. Margeat and S. Weiss, *Proceedings of the National Academy of Sciences*, 2004, **101**, 8936-8941.
6. A. Ingargiola, E. Lerner, S. Chung, F. Panzeri, A. Gulinatti, I. Rech, M. Ghioni, S. Weiss and X. Michalet, *PLoS One*, 2017, **12**, e0175766.
7. G. Agam, C. Gebhardt, M. Popara, R. Mächtel, J. Folz, B. Ambrose, N. Chamachi, S. Y. Chung, T. D. Craggs, M. de Boer, D. Grohmann, T. Ha, A. Hartmann, J. Hendrix, V. Hirschfeld, C. G. Hübner, T. Hugel, D. Kammerer, H.-S. Kang, A. N. Kapanidis, G. Krainer, K. Kramm, E. A. Lemke, E. Lerner, E. Margeat, K. Martens, J. Michaelis, J. Mitra, G. G. Moya Muñoz, R. B. Quast, N. C. Robb, M. Sattler, M. Schlierf, J. Schneider, T. Schröder, A. Sefer, P. S. Tan, J. Thurn, P. Tinnefeld, J. van Noort, S. Weiss, N. Wendler, N. Zijlstra, A. Barth, C. A. M. Seidel, D. C. Lamb and T. Cordes, *Nature Methods*, 2023, **20**, 523-535.
8. T. Förster, *Annalen der Physik*, 1948, **437**, 55-75.
9. R. S. Mathew-Fenn, R. Das, J. A. Silverman, P. A. Walker and P. A. Harbury, *PLoS One*, 2008, **3**, e3229.
10. T. Narayanan, M. Sztucki, T. Zinn, J. Kieffer, A. Homs-Puron, J. Gorini, P. Van Vaerenbergh and P. Boesecke, *Journal of Applied Crystallography*, 2022, **55**, 98-111.
11. M. Sztucki, E. Di Cola and T. Narayanan, *The European Physical Journal Special Topics*, 2012, **208**, 319-331.
12. M. Sztucki, E. Di Cola and T. Narayanan, *Journal of Applied Crystallography*, 2010, **43**, 1479-1487.
13. T. Zettl, R. S. Mathew, S. Seifert, S. Doniach, P. A. B. Harbury and J. Lipfert, *Nano Letters*, 2016, **16**, 5353-5357.
14. V. J. Pinfield and D. J. Scott, *PLoS One*, 2014, **9**, e95664.
15. S. F. Gull and J. Skilling, *Maximum Entropy Data Consultants Ltd., Suffolk*, 1999, 1-108.
16. A. Guinier, *Annales de physique*, 1939, **Vol. 11**, pp. 161-237.
17. D. Svergun, C. Barberato and M. H. J. Koch, *Journal of Applied Crystallography*, 1995, **28**, 768-773.
18. C. Gebhardt, M. Lehmann, M. M. Reif, M. Zacharias, G. Gemmecker and T. Cordes, *Chemphyschem*, 2021, **22**, 1566-1583.
19. P. D. Jadzinsky, G. Calero, C. J. Ackerson, D. A. Bushnell and R. D. Kornberg, *Science*, 2007, **318**, 430-433.

20. E. Gasteiger, C. Hoogland, A. Gattiker, S. E. Duvaud, M. R. Wilkins, R. D. Appel and A. Bairoch, Humana Press, 2005, DOI: 10.1385/1-59259-890-0:571, pp. 571-607.
21. P. G. Telmer and B. H. Shilton, *Journal of Biological Chemistry*, 2003, **278**, 34555-34567.
22. B. H. Shilton, M. M. Flocco, M. Nilsson and S. L. Mowbray, *Journal of Molecular Biology*, 1996, **264**, 350-363.
23. A. J. Sharff, L. E. Rodseth, J. C. Spurlino and F. A. Quiocho, *Biochemistry*, 1992, **31**, 10657-10663.
24. F. A. Quiocho, J. C. Spurlino and L. E. Rodseth, *Structure*, 1997, **5**, 997-1015.
25. T. Zettl, R. Das, P. A. B. Harbury, D. Herschlag, J. Lipfert, R. S. Mathew and X. Shi, *Curr Protoc Nucleic Acid Chem*, 2018, **73**, e54.
26. M. M. Tirion, *Physical Review Letters*, 1996, **77**, 1905-1908.
27. R. Machtel, A. Narducci, D. A. Griffith, T. Cordes and C. Orelle, *Res Microbiol*, 2019, **170**, 321-337.
28. T. Ferenci, M. Muir, K.-S. Lee and D. Maris, *Biochimica Et Biophysica Acta (BBA)-Biomembranes*, 1986, **860**, 44-50.
